# β-Carboline Alkaloids From the Deep-Sea Fungus *Trichoderma* sp. MCCC 3A01244 as a New Type of Anti-pulmonary Fibrosis Agent That Inhibits TGF-β/Smad Signaling Pathway

**DOI:** 10.3389/fmicb.2022.947226

**Published:** 2022-07-28

**Authors:** Meng-Jiao Hao, Pei-Nan Chen, Hou-Jin Li, Feng Wu, Guang-Yu Zhang, Zong-Ze Shao, Xiu-Pian Liu, Wen-Zhe Ma, Jun Xu, Taifo Mahmud, Wen-Jian Lan

**Affiliations:** ^1^School of Pharmaceutical Sciences, Sun Yat-sen University, Guangzhou, China; ^2^School of Chemistry, Sun Yat-sen University, Guangzhou, China; ^3^Key Laboratory of Marine Biogenetic Resources, Third Institute of Oceanography, Ministry of Natural Resources, Xiamen, China; ^4^State Key Laboratory of Quality Research in Chinese Medicine, Macau University of Science and Technology, Macau, Macau SAR, China; ^5^Department of Pharmaceutical Sciences, Oregon State University, Corvallis, OR, United States

**Keywords:** β-carboline alkaloids, *Trichoderma*, acid-directed strategy, anti-pulmonary fibrosis, TGF-β/Smad

## Abstract

Pulmonary fibrosis is a scarring disease of lung tissue, which seriously threatens human health. Treatment options are currently limited, and effective strategies are still lacking. In the present study, 25 compounds were isolated from the deep-sea fungus *Trichoderma* sp. MCCC 3A01244. Among them, two β-carboline alkaloids, trichocarbolines A (**1**) and C (**4**) are new compounds. The chemical structures of these compounds were elucidated based on their HRESIMS, 1D and 2D NMR spectra, optical rotation calculation, and comparisons with data reported in the literature. Trichocarboline B [(+)- and (–)-enantiomers] had previously been synthesized, and this is its first report as a natural product. Their anti-pulmonary fibrosis (PF) activity and cytotoxicity were investigated. Compounds **1**, **11**, and **13** strongly inhibited TGF-β1-induced total collagen accumulation and showed low cytotoxicity against the HFL1 cell line. Further studies revealed compound **1** inhibited extracellular matrix (ECM) deposition by downregulating the expression of protein fibronectin (FN), proliferating cell nuclear antigen (PCNA), and α-smooth muscle actin (α-SMA). Mechanistic study revealed that compound **1** decreased pulmonary fibrosis by inhibiting the TGF-β/Smad signaling pathway. As a newly identified β-carboline alkaloid, compound **1** may be used as a lead compound for developing more efficient anti-pulmonary fibrosis agents.

## Introduction

Damage to alveolar epithelial cells, excessive proliferation of fibroblasts, and inappropriate deposition of extracellular matrix (ECM) produce pulmonary fibrosis (PF), which leads to scarring, impaired lung function, and ultimately lung failure (Herrera et al., 2018). At least five million people are affected by pulmonary fibrosis globally, and the average life expectancy for people with pulmonary fibrosis is less than 5 years (Lynch and Belperio, 2012). Pulmonary fibrosis is the main manifestation of the sequelae of COVID-19 (Zhou et al., 2021). PF is estimated to occur in about one-third of patients hospitalized with COVID-19 as of July 2020 (Vasarmidi et al., 2020). To date, two available antifibrotic drugs, pirfenidone and nintedanib have been approved by FDA for treating idiopathic pulmonary fibrosis (IPF). However, clinical application of nintedanib is limited due to poor oral bioavailability, metabolic instability, and off-target side effects (Roth et al., 2015). Treatment with pirfenidone can produce skin and gastrointestinal-related adverse effects (Cottin and Maher, 2015). Hence, more effective and safer drugs for pulmonary fibrosis treatment are urgently needed.

The master target for antifibrotic therapies is the TGF-β pathway. TGF-β is upregulated and activated in fibrotic diseases. TGF-β1 triggers a pro-fibrotic response *via* activation of the Smad-2/3 cascade, which regulates fibroblast phenotype and function, induces myofibroblast transdifferentiation, and promotes ECM deposition (Biernacka et al., 2011). The intervention of the intracellular phosphorylation of Smad-2/3 protein can reduce TGF-β-induced fibrosis (Walton et al., 2017). Thus, exogenous compounds that disrupt TGF-β/Smad signaling and inhibit myofibroblast activation are likely to be potential anti-pulmonary fibrosis drugs.

As part of our efforts to discover new natural products with anti-pulmonary fibrosis activity, we investigated the chemical constituents of a fungal strain, *Trichoderma* sp. MCCC 3A01244, collected at the 3300 m depth in the Northern Basin of the South China Sea. *Trichoderma* species are commonly found in diverse environments (Reino et al., 2008). Fungi from this genus can produce a variety of structurally intriguing compounds, including terpenoids, polyphenols, pyrones, cyclopeptides, and polyketides (Tchameni et al., 2020). Many of them showed various biological activities, including antimicrobial (Shi et al., 2020), antimicroalgal (Zou et al., 2021a), antioxidant (Miyano et al., 2020), antifouling (Yu et al., 2021), anti-hepatitis C virus (HCV) (Li B. et al., 2019), and cytotoxic activities (Liu et al., 2020), implying the potential of *Trichoderma* species as a source of drugs for agricultural and/or human uses. Some *Trichoderma* species have been commercialized as agents to control phytopathogenic fungi or stimulate plant growth (Morán-Diez et al., 2021; Zou et al., 2021b). Here, we report the isolation, structure characterization, anti-PF activity, and cytotoxicity of secondary metabolites isolated from the deep-sea fungus *Trichoderma* sp. MCCC 3A01244. Among them, trichocarboline A (**1**), a β-carboline alkaloid, is potentially anti- pulmonary fibrosis by inhibiting TGF-β/Smad signaling pathway.

## Materials and Methods

### General Experimental Procedures

Optical rotations were measured on an Anton Paar MCP500 polarimeter. IR spectra were obtained on a Bruker Tensor-27 spectrophotometer. UV spectra were measured by a Shimadzu UV-vis-NIR spectrophotometer. NMR spectra were acquired on Bruker Avance II 400 and 500 spectrometers (Bruker Bio Spin AG, Industriestrasse 26, Fallanden, Switzerland). The chemical shifts are referred to the residual solvent signals (acetone-*d*_6_: δ_*H*_ 2.05, δ_*C*_ 29.8; CDCl_3_: δ_*H*_ 7.26, δ_*C*_ 77.2; CD_3_OD: δ_*H*_ 3.30, δ_*C*_ 49.0; DMSO-*d*_6_: δ_*H*_ 2.50, δ_*C*_ 39.5). HRESIMS data were recorded on Thermo DSQ EI low-resolution and Thermo MAT95XP EI high-resolution mass spectrometers (Thermo Fisher Scientific Inc.). Silica gel (200-300 mesh, Qingdao Marine Chemical Factory) and Sephadex LH-20 (GE Healthcare) were used for column chromatography. Preparative HPLC adopted a Shimadzu LC-20AT HPLC pump (Shimadzu Corporation, Nakagyo-Ku, Kyoto, Japan) with an SPD-20A dual λ absorbance detector (Shimadzu Corporation, Nakagyo-Ku, Kyoto, Japan), as well as a Shim-pack PRC-ODS HPLC column (250 × 20 mm, Shimadzu Corporation, Nakagyo-Ku, Kyoto, Japan) and a Chiral CD-Ph HPLC column (250 × 10 mm, Shimadzu Corporation, Nakagyo-Ku, Kyoto, Japan).

### Fungal Material and Fermentation

The deep-sea fungus *Trichoderma* sp. MCCC 3A01244 was obtained from the Marine Culture Collection of China (MCCC). It was originally separated from seawater at the depth of 3300 m in the northern basin of the South China Sea. It was persevered in the School of Pharmaceutical Sciences, Sun Yat-sen University, Guangzhou, China. This fungal strain was identified according to the morphological characteristics and analysis of internal transcribed spacer (ITS) rDNA. The ITS gene sequence was deposited in NCBI’s GenBank with the accession number MW581838. The fermentation medium consists of glucose 10 g/L, peptone 5 g/L, yeast extract 2 g/L, L-Trp 3 g/L, L-Ser 2 g/L, L-Thr 2 g/L, L-Lys 2 g/L, L-Phe 2 g/L, L-Val 2 g/L, L-Met 2 g/L, sea salt 20 g/L and water 1 L (pH adjusted to 7.0). Fungal mycelia were crumbled and transferred aseptically to Erlenmeyer flasks. The flasks, each containing 400 mL sterilized liquid medium, were statically incubated at 28 °C for 30 days.

### Extraction and Isolation

After 30 days of fermentation, the culture broth and the mycelia (200 L) were separated by filtration and extracted exhaustively with EtOAc and MeOH, respectively. The EtOAc extract was evaporated to afford a crude extract (69 g). The MeOH extract was concentrated *in vacuo* to yield an oily brown residue (19 g). The EtOAc extract was then subjected to column chromatography (CC) over silica gel with a gradient of petroleum ether–EtOAc (10:0-0:10) to EtOAc–MeOH (10:0-0:10) to afford 7 fractions (Fr.1–Fr.7). Fr.3 was subsequently separated by Sephadex LH-20 (MeOH) to provide five subfractions (Fr.3.1–Fr.3.5). Fr.3.2 was further fractionated by preparative HPLC with MeOH–H_2_O (65:35 v/v) to yield compounds **14** (37.1 mg) and **23** (26.9 mg). Fr.3.4 was chromatographed by preparative HPLC with MeOH–H_2_O (43:57 v/v) to afford **8** (16.4 mg) and **16** (10.5 mg). Compounds **10** (3.0 mg) and **11** (5.0 mg) were obtained from Fr.4 by chromatography on a Sephadex LH-20 column (MeOH) and then on a preparative HPLC column (MeOH–H_2_O, 78:22 v/v). Compounds **6** (8.7 mg), **20** (7.8 mg) and **22** (30.1 mg) were also purified from Fr.4 using preparative HPLC with MeOH–H_2_O (55:45 v/v). Fr.5 was subdivided to five subfractions (Fr.5.1–Fr.5.5) using a silica gel column with a stepwise gradient of petroleum ether–EtOAc (10:0–0:10). Fr.5.2 was separated by Sephadex LH-20 CC (MeOH) to give compound **15** (20.8 mg). Compounds **1** (3.0 mg) and **13** (1.0 mg) were isolated from Fr.5.4 by Sephadex LH-20 CC (MeOH). Fr.5.3 was fractionated by a silica gel column and a preparative HPLC to give compounds **19** (8.9 mg), **21** (21.0 mg), **5** (2.0 mg), **7** (10.0 mg) and the mixture of **2** and **3** (10 mg). Fr.6 was separated by repeated CC on a silica gel column and Sephadex LH-20 (MeOH) to afford compounds **4** (0.8 mg) and **17** (2.4 mg).

On the other hand, the MeOH extract was subjected to a silica gel column with a gradient of petroleum ether–EtOAc (10:0–0:10) to EtOAc–MeOH (10:0–0:10) to afford 12 fractions (Fr.M-1–Fr.M-12). Compound **12** (22.5 mg) was obtained from Fr.M-7 by chromatography on a Sephadex LH-20 column (MeOH) followed by a preparative HPLC column (MeOH–H_2_O, 75:25 v/v). Fr.M-11 was subjected to Sephadex LH-20 CC (MeOH) and subsequently separated by preparative HPLC (MeOH–H_2_O, 60:40 v/v) to obtain **24** (2.0 mg) and **25** (2.0 mg). Similarly, Fr.M-12 was separated by Sephadex LH-20 CC (MeOH) and purified by preparative HPLC (MeOH–H_2_O, 33:67 v/v) to give compounds **9** (5.3 mg) and **18** (1.3 mg).

### Spectroscopic Data

*Trichocarboline A (**1**)*: a light yellow powder, [α]20 D -29.0 (*c* 0.05, MeOH); UV (MeOH) λ_*max*_ (log ε) 283 (4.33), 305 (4.05), 378 (3.99); IR: ν_*max*_ 3326, 2918, 2849, 1671, 1646, 1626, 1469, 1433, 1322, 1204, 1128, 1062, 1015 cm^–1^; ^1^H and ^13^C NMR data see Table 1; HR(-)ESIMS *m/z* 283.1089 [M - H]^–^ (calcd for C_16_H_15_N_2_O_3_, 283.1088).

**TABLE 1:** ^1^H (400 MHz) and ^13^C NMR (101 MHz) data for compounds **1**, **2/3**, and **4** (δ in ppm, *J* in Hz).

	1 (in CD_3_OD)		2/3 (in CD_3_OD)		4 (in CDCl_3_)	
Position	δ_*C*_, type	δ_*H*_, mult. (*J* in Hz)	δ_*C*_, type	δ_*H*_, mult. (*J* in Hz)	δ_*C*_, type	δ_*H*_, mult. (*J* in Hz)
1	137.1, C		148.5, C		135.1, C	
3	138.5, CH	8.42, d (4.8)	137.3, CH	8.21, d (5.6)	138.5, CH	8.01, d (4.8)
4	120.1, CH	8.26, d (4.8)	114.8, CH	7.99, d (5.6)	118.1, CH	8.47, d (4.8)
5	122.7, CH	8.18, d (7.6)	122.4, CH	8.17, d (8.0)	123.1, CH	7.97, d (8.4)
6	121.6, CH	7.27, dd (7.6, 7.2)	120.7, CH	7.25, dd (8.0, 7.2)	110.6, CH	6.85, d (8.4)
7	130.3, CH	7.55, dd (8.0, 7.2)	129.6, CH	7.55, dd (8.0, 7.2)	157.8, C	
8	113.5, CH	7.66, d (8.0)	113.0, CH	7.63, d (8.0)	97.7, CH	6.98, s
9	NH		NH		NH	10.21, brs
10	136.2, C		134.5, C		136.0, C	
11	133.2, C		131.2, C		131.9, C	
12	121.7, C		122.1, C		114.4, C	
13	143.4, C		142.6, C		143.1, C	
1’	204.9, CO		76.5, CH	5.09, t (6.4)	206.0, CO	
2’	35.0, CH_2_	3.46, t (7.6)	31.0, CH_2_	2.01, m	31.2, CH_2_	3.42, q (7.2)
3’	29.0, CH_2_	1.83, m 2.04, m	10.2, CH_3_	0.99, t (7.2)	8.3, CH_3_	1.30, t (7.2)
4’	72.8, CH	3.72, m				
5’	67.4, CH_2_	3.52, m				

(–)*-Trichocarboline B (**2**)*: a light yellow powder, [α]20 D -106.0 (*c* 0.10, MeOH); UV (MeOH) λ_*max*_ (log ε) 235 (4.84), 289 (4.49), 339 (4.02), 349 (4.01) nm; IR: ν_*max*_ 3557, 2962, 2925, 2873, 1627, 1567, 1494, 1456, 1430, 1323, 1238, 1045 cm^–1^; ^1^H and ^13^C NMR data see Table 1; HR(-)ESIMS *m/z* 225.1034 [M - H]^–^ (calcd for C_14_H_13_N_2_O, 225.1033).

(+)*-Trichocarboline B (**3**)*: a light yellow powder, [α]20 D + 100.0 (*c* 0.08, MeOH); UV (MeOH) λ_*max*_ (log ε) 235 (4.84), 289 (4.49), 339 (4.02), 349 (4.01) nm; IR: ν_*max*_ 3557, 2962, 2925, 2873, 1627, 1567, 1494, 1456, 1430, 1323, 1238, 1045 cm^–1^; ^1^H and ^13^C NMR data see Table 1; HR(-)ESIMS *m/z* 225.1034 [M - H]^–^ (calcd for C_14_H_13_N_2_O, 225.1033).

*Trichocarboline C (**4**)*: a red solid; UV (MeOH) λ_*max*_ (log ε) 217 (2.37) nm; IR: ν_*max*_ 3398, 2946, 1726, 1446, 1354, 1320, 1185, 1024, 958, 875 cm^–1^; ^1^H and ^13^C NMR data see Table 1; HR(+)ESIMS *m/z* 241.0978 [M + H]^+^ (calcd for C_14_H_13_N_2_O_2_, 241.0972).

### Chiral Separation of 2 and 3

By using a Chiral CD-Ph column (MeOH/H_2_O 60:40; flow rate 1.0 mL/min), the mixture of enantiomers **2** and **3** was resolved to afford **2** (3.0 mg, *t*_*R*_ = 32.5 min) and **3** (3.0 mg, *t*_*R*_ = 40.0 min).

### Specific Optical Rotation Calculation

The specific optical rotation values of compounds **1**-**3** were calculated by quantum chemical calculations using Gaussian 09 software (Li et al., 2012). They were further optimized by the density functional theory method at the B3LYP/6-311G (2d, p) level and calculations were made at the PBE1PBE/6-311 + + G (d, p) level in MeOH with a PCM model. The calculated specific optical rotation was averaged according to the Boltzmann distribution theory and their relative Gibbs free energy.

### Cell Culture and Cytotoxicity Assays

The human fetal lung fibroblasts (HFL1) were purchased from Procell Life Science and Technology Co., Ltd (Cat No.: CL-0106 Wuhan, China). Cells were cultured in Ham’s F-12K medium (PM150910, Procell Life Science and Technology, Wuhan) supplemented with 10% fetal bovine serum (FBS) (#10270-106, GIBCO, Invitrogen, Carlsbad, CA, United States) and 1% penicillin-streptomycin in an incubator at 37°C with 5% CO_2_. According to the manufacturer’s protocol, the cell viability was measured using the Cell Counting Kit-8 (CCK8). The cells were seeded in 96-well plates at a density of 5 × 10^3^ cells/well. After incubating for 24 h, the cells were treated with a medium containing 10 μM compounds **1**-**3**, **5**-**25**, pirfenidone (TargetMol, United States) for 48 h. Following incubation, each well was incubated at 37°C for 2 h with 10 μL of CCK8 solution. After that, a full function microplate reader (BioTek, United States) was used to measure the solution’s absorbance at 450 nm. Survival rate = (A value, Administration)/(A value, Control) × 100%. All assays were repeated in triplicate.

### Inhibition of Collagen Accumulation Rate *in vitro*

The antifibrosis activities of the compounds were investigated in HFL1 cells seeded in 96-well plates at a density of 2 × 10^4^ cells/well. After incubation for 24 h, the cells were treated with a medium containing TGF-β1 (5 ng/mL) and 10 μM compounds **1**-**3**, **5**-**25**, pirfenidone for 48 h. Afterward, the supernatant was removed, and the cells were fixed for 30 min with 4% paraformaldehyde. After washing twice with PBS, the cells were added the 0.1% Sirius red dye with saturated picric acid. After 4 h of staining protected from light, the collagenous fiber was dyed red. Then, the cells were washed three times with 0.1% acetic acid and visualized under the microscope cell imaging system (EVOS FL Auto, Life Technologies, United States). For the quantitative determinations of the accumulated collagen, the stained cells were destained with 0.1M NaOH (100 μL/well) for 10 min. Then, the absorbance was measured at 540 nm with a spectrophotometer. Total collagen accumulation inhibition = 1 - (Administration A value - control A value)/(model A value - control A value) × 100%. All assays were repeated in triplicate (Xue et al., 2020).

### Western Blot Analysis

Western blot analysis was performed as previously described methods (Hao et al., 2020). The primary antibodies: anti-α-SMA (Cat No. Ab7817), anti-fibronectin (Cat No. 15613-1-AP), anti-PCNA (Cat No. 10205-2-AP), anti-phospho-Smad2^*Ser*255^ (Cat No. Ab188334), anti-phospho-Smad3^*Ser*423/425^ (Cat No. Ab52903), anti-Smad2 (Cat No. Ab40855), anti-Smad3 (Cat No. Ab40854) and anti-GAPDH (Cat No. 10494-1-AP).

### Statistical Analysis

Data are expressed as the means ± SEM. The GraphPad Prism 6.0 software (San Diego, CA, United States) was used to perform statistical analysis. The one-way analysis of variance (ANOVA) and *post-hoc* test (LSD) were used to analyze the significant differences between groups. All differences were considered statistically significant at *P* < 0.05.

## Results

### Isolation and Structure Elucidation

**FIGURE 1:**
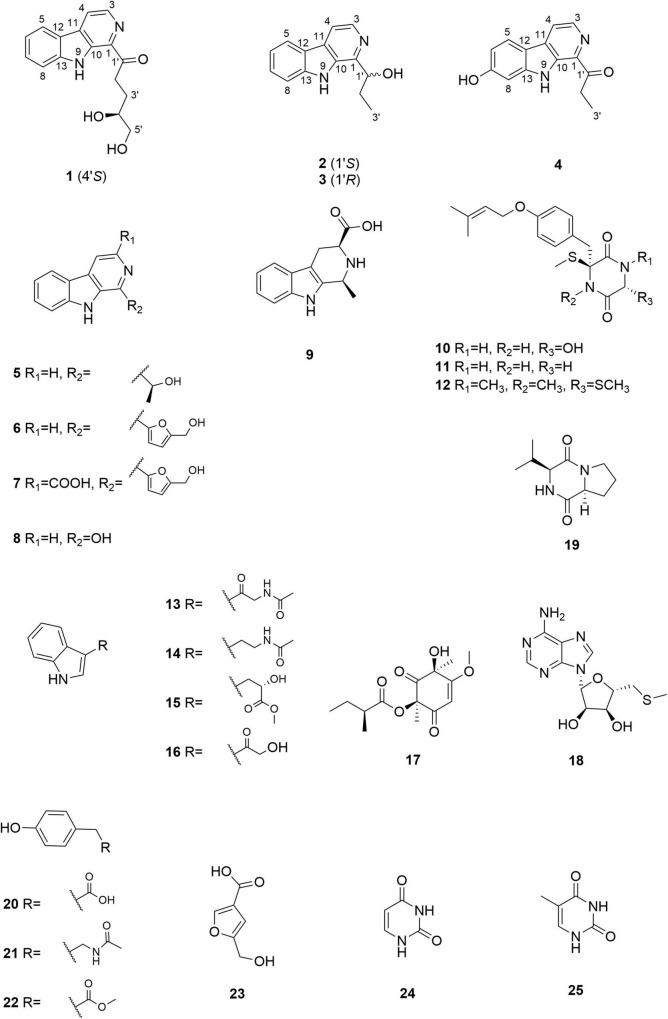
Chemical structures of compounds **1**–**25**.

To induce the production of secondary metabolites in the MCCC 3A01244 strain, we employed the amino acid–directed strategy (Huang et al., 2017). The fungus was grown in GYP medium supplemented with L-Trp 2 g/L, L-Ser 2 g/L, L-Thr 2 g/L, L-Lys 2 g/L, L-Phe 2 g/L, L-Val 2 g/L, and L-Met 2 g/L. The culture was statically incubated at 28 °C for 30 days, at which point the mycelia and the culture broth were separated by filtration and extracted exhaustively with MeOH and EtOAc, respectively. The extracts were subsequently subjected to successive column chromatography and HPLC. Consequently, 25 structurally diverse natural products were identified from the extracts (Figure 1), including four β-carbolines, trichocarbolines A, B [(+)- and (–)-enantiomers], and C (**1**-**4**).

**FIGURE 2:**
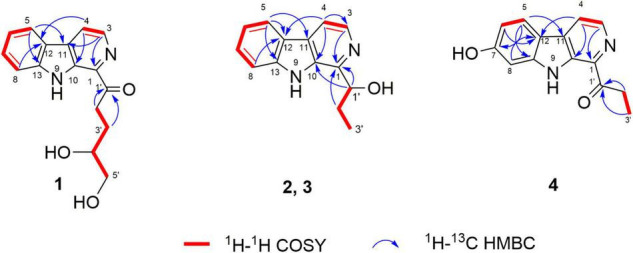
Key COSY and HMBC correlations of compounds **1**–**4**.

Trichocarboline A (**1**) was isolated as a light-yellow powder. The molecular formula of **1** was established as C_16_H_16_N_2_O_3_ according to the HR(-)ESIMS ion at *m*/*z* 283.1089 [M-H]^–^ (calcd 283.1088 for C_16_H_15_N_2_O_3_), indicating ten degrees of unsaturation. The ^13^C NMR spectrum, in combination with DEPT-135 and HSQC spectra (Table 1), showed resonances for three sp^3^ methylenes (including one oxygenated methylene), six sp^2^ methines, one oxygenated sp^3^ methine, five non-protonated sp^2^ carbons, and one carbonyl carbon. The ^1^H NMR spectrum of **1** showed resonances at δ_*H*_ 8.18 (1H, d, *J* = 7.6 Hz, H-5), 7.27 (1H, dd, *J* = 7.6, 7.2 Hz, H-6), 7.55 (1H, dd, *J* = 8.0, 7.2 Hz, H-7), and 7.66 (1H, d, *J* = 8.0 Hz, H-8) in the ^1^H NMR spectrum, which along with the ^1^H-^1^H COSY correlations of H-5/H-6/H-7/H-8 revealed the presence of an *ortho*-substituted benzene ring. Additionally, the COSY spectrum also indicated the presence of a pair of aromatic protons at δ_*H*_ 8.42 (1H, d, *J* = 4.8 Hz, H-3) and 8.26 (1H, d, *J* = 4.8 Hz, H-4). The HMBC correlations from H-3 to C-1 (δ_*C*_ 137.1) and C-11 (δ_*C*_ 133.2), from H-4 to C-10 (δ_*C*_ 136.2) and C-12 (δ_*C*_ 121.7), from H-5 to C-11 and C-13 (δ_*C*_ 143.4), and from H-8 to C-12 established a β-carboline moiety. Moreover, from the ^1^H − ^1^H COSY correlations of H-2′ (δ_*H*_ 3.46, t)/H-3′ (δ_*H*_ 2.04, m; 1.83, m), H-3′/H-4′ (δ_*H*_ 3.72, m) and H-4′/H-5′ (δ_*H*_ 3.52, m), the fragment of –CH_2_CH_2_CH(OH)CH_2_OH was postulated. The HMBC correlations from H-2′/H-3′ to C-1′ (δ_*C*_ 204.9) demonstrated that the carbonyl group is linked with C-2′ (δ_*C*_ 35.0). Although no HMBC correlation was observed to connect C-1 with C-1′, the overall NMR data for **1** as well as direct comparisons of the ^1^H and ^13^C NMR spectra of **1** with those of **2** and **3** (see below), strongly suggest that the side chain is connected to C-1. The absolute configuration of the hydroxy group at C-4′ was determined to be *S*, as the calculated optical rotation value of 4′*S*-**1** (-32.9) fitted well with the experimental data for **1** (-29.0). Accordingly, the structure of trichocarboline A (**1**) was established as shown in Figure 1.

(–)- and (+)-Trichocarbolines B (**2** and **3**, respectively) were initially obtained as a mixture of enantiomers (a yellow powder) and their molecular formula was established as C_14_H_14_N_2_O based on HR(-)ESIMS ion at *m*/*z* 225.1034 [M-H]^–^ (calcd 225.1033 for C_14_H_13_N_2_O), corresponding to nine degrees of unsaturation. The ^13^C NMR and DEPT-135 spectra of **2** and **3** displayed resonances for 11 aromatic carbons similar to those of **1** (Table 1), suggesting the presence of a β-carboline skeleton. The key HMBC correlations from H-4 (1H, δ_*H*_ 7.99, d, *J* = 5.6 Hz) to C-10 (δ_*C*_ 134.5) and C-12 (δ_*C*_ 122.1), and from H-5 (1H, δ_*H*_ 8.17, d, *J* = 7.6 Hz) to C-11 (δ_*C*_ 131.2) and C-13 (δ_*C*_ 142.6) along with the ^1^H-^1^H COSY correlations between H-3 (1H, δ_*H*_ 8.21, d, *J* = 5.6 Hz) and H-4, between H-5 and H-6 (1H, δ_*H*_ 7.25, dd, *J* = 7.6, 7.2 Hz), between H-6 and H-7 (1H, δ_*H*_ 7.54, dd, *J* = 8.0, 7.2 Hz), and between H-7 and H-8 (1H, δ_*H*_ 7.63, d, *J* = 8.0 Hz) further corroborated the structure of a β-carboline moiety (Figure 2). The ^1^H−^1^H COSY cross-peaks of H-1′ (1H, δ_*H*_ 5.09, t, *J* = 6.4 Hz) and H-2′ (2H, δ_*H*_ 2.01, m), H-2′ and H-3′ (3H, δ_*H*_ 0.99, t, *J* = 7.2 Hz) and the HMBC correlations of H-1′/C-1 and H-2′/C-1 (δ_*C*_ 148.8) (Figure 2) revealed the presence of a –CH(OH)CH_2_CH_3_ side chain, which is connected to C-1. Based on these data, the enantiomeric mixture was identified as 1-(9*H*-pyrido[3,4-*b*]indol-1-yl)propane-1-ol, which was reported recently as a synthetic product (Szepesi Kovács et al., 2021), but no enantiomeric purity was determined. Since the enantiomers often have disparate pharmacological activities and even metabolic pathways (Jiao et al., 2015), we decided to separate the enantiomers for further biological evaluation. After several attempts using a diverse set of chiral LC columns, we were able to separate the two enantiomers (–)-trichocarboline B (**2**) and (+)-trichocarboline B (**3**) and assigned their absolute configurations by comparing their optical rotations with the calculated values for the 2′*S* and 2′*R* isomers. The experimental optical rotation value of the faster eluting enantiomer (**2**) was -106.0 and the calculated optical rotation value of 2′*S* was -97.4, suggesting that the absolute configuration of **2** is 2′*S*. The calculated optical rotation for 2′*R* was + 97.3, which matched well with the experimental value of the second eluting enantiomer **3** (+100.0).

Compound **4**, named trichocarboline C, was found as a red powder. The molecular formula of **4** was determined to be C_14_H_12_N_2_O_2_ based on the HR(+)ESIMS protonated ion peak at *m/z* 241.0978 [M + H]^+^ (calcd for C_14_H_13_N_2_O_2_, 241.0972). The ^13^C NMR spectrum, in conjunction with DEPT and HSQC spectra (Table 1), showed fourteen carbon signals including one sp^3^ methyl (δ_*C*_ 8.3), one sp^3^ methylene (δ_*C*_ 31.2), five sp^2^ methines (δ_*C*_ 97.7, 110.6, 118.1, 123.1, 138.5), six non-protonated sp^2^ carbons (δ_*C*_ 114.4, 131.9, 135.1, 136.0, 138.5, 143.1), and one carbonyl carbon (δ_*C*_ 206.0). The ^1^H − ^1^H COSY correlations between H-3 (1H, δ_*H*_ 8.01, d, *J* = 4.8 Hz) and H-4 (1H, δ_*H*_ 8.47, d, *J* = 4.8 Hz), between H-5 (1H, δ_*H*_ 7.97, d, *J* = 8.4 Hz) and H-6 (1H, δ_*H*_ 6.85, d, *J* = 8.4 Hz) and between H-2′ (2H, δ_*H*_ 3.42, q, *J* = 7.2 Hz) and H-3′ (3H, δ_*H*_ 1.30, t, *J* = 7.2 Hz), as well as HMBC correlations from H-3 to C-1 (δ_*C*_ 135.1), from H-4 to C-12 (δ_*C*_ 114.4) and C-10 (δ_*C*_ 136.0), from H-5 to C-11 (δ_*C*_ 131.9), C-7 (δ_*C*_ 157.8) and C-13 (δ_*C*_ 143.1) and from H-6 and H-8 (δ_*H*_ 6.98, s) to C-12 (δ_*C*_ 114.4) (Figure 2) indicated that **4** possesses the same β-carboline core structure as trichocarboline A (**1**). Moreover, the ^1^H−^1^H COSY correlation between H-2′ and H-3′ and HMBC correlations from H-2′/H-3′ to C-1′ (δ_*C*_ 206.0) indicated the presence of a propionyl group, which, based on comparisons of its NMR data with those of compounds **1**–**3**, was postulated to be connected to C-1 (Figure 2). Furthermore, the assignment of a hydroxyl group at C-7 (δ_*C*_ 157.8) was based on a combination of the ^1^H−^1^H COSY and HMBC correlations shown in Figure 2. Consequently, the chemical structure of **4** was elucidated as depicted in Figure 1.

In addition to the four compounds described above, we also isolated 21 known compounds, i.e., cordysinin C (**5**) (Yang et al., 2006), perlolyrine (**6**) (Santhanam et al., 2020), flazine (**7**) (Santhanam et al., 2020), 3-hydroxy-β-carboline (**8**) (Jiao et al., 2010), 1,2,3,4-tetrahydro-1-methyl-β-carboline-3-carboxylic acid (**9**) (Kicha et al., 2003), 6-hydroxy-3-methylthio-3-[4′-(3′′-methyl-2′′-butenoxy) phenylmethyl]-2,5-piperazinedione (**10**) (Ayer et al., 1990), 3-thiomethyl-3-[4′′-(3′′-methyl-2′′-butenoyl)phenylmethyl]-2,5-piperazinedione (**11**) (Ayer et al., 1990), bis(methylthio)silvatin (**12**) (Wang et al., 1998), *N*-acetyl-β-oxotryptamine (**13**) (Yang et al., 2013), *N*-[2-(1*H*-indol-3-yl)ethyl]acetamide (**14**) (Häring et al., 2017), indole-3-lactic acid methyl ester (**15**) (Nguyen et al., 2010), 3-(2-hydroxyacetyl)indole (**16**) (Pettit et al., 2006), phomaligol A (**17**) (Li et al., 2003), 5′-deoxy-5′-methylthioadenosine (**18**) (Jiao et al., 2019), cyclo-L-prolyl-L-valine (**19**) (Begum Ahil et al., 2019), *N*-acetyltyramine (**20**) (Lee et al., 2017), 4-hydroxyphenylacetate (**21**) (Davis et al., 2011), methyl 4-hydroxyphenylacetate (**22**) (Qiu et al., 2017), 5-(hydroxymethyl)-3-furancarboxylic acid (**23**) (Evidente et al., 2009), uracil (**24**) (Kan et al., 2011), and thymine (**25**) (Kuchkarova et al., 2020). All of these compounds were identified by comparing their ^1^H and ^13^C NMR data (Supplementary Figures 22–63) with those reported in the literatures.

### Proposed Biosynthetic Pathway

The putative biosynthetic pathway for β-carboline alkaloids has been described in the previous paper published by our team (Qiu et al., 2020). Briefly, compound **1** was supposed to be biosynthesized *via* the McbB enzymatic Pictet-Spengler reaction with tryptamine and glucose, using the negatively charged Glu97 to complete the aromatization, followed by consecutive decarboxylation and oxidation (Chen et al., 2013; Chen et al., 2018; Qiu et al., 2020). Similarly, compounds **2**-**5** were also generated from tryptophan and corresponding aldehydes. Detailed descriptions of the hypothetical biosynthetic pathway for compounds **6**-**7** have been disclosed in a previous study (Zhao et al., 2017).

### Preliminary Screening of Compounds 1–25 for Inhibiting Collagen Accumulation

**FIGURE 3:**
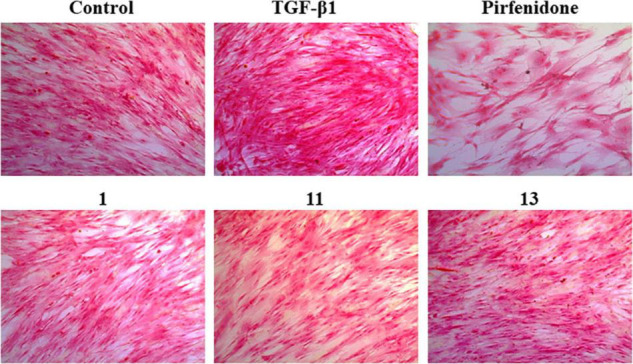
Picro-Sirius Red (PSR) staining for the total collagen accumulation induced by TGF-β1 in HFL1 cells. The representative pictures are the cells induced by TGF-β1 and treated with 10 μM compounds **1**, **11**, and **13** pirfenidone, and the control group (untreated normal cells).

The pathological marker of fibrosis is the abnormal deposition of excessive extracellular matrix (ECM) with collagen as the main component. Therefore, the detection of collagen synthesis, which can be directly correlated to the degree of fibrosis, is an effective indicator for evaluating fibrotic diseases. Trichocarboline A (**1**), (–)- and (+)-trichocarbolines B (**2** and **3**), together with 21 compounds (**5**–**25**) were preliminarily screened for their cytotoxicity in HFL1 cells at a concentration of 10 μM using a Cell Counting Kit-8 (CCK8)-based assay. Trichocarboline D (**4**) was not evaluated for its activity due to insufficient quantity. The Sirius red dye staining, which has been accepted to be an effective and convenient method for the anti-fibrotic screening model *in vitro* (Deng et al., 2020; Xue et al., 2020), was then used to evaluate compound inhibitory activity on total collagen accumulation induced by TGF-β1. Pirfenidone was used as a positive control. As illustrated in Table 2, compounds **1**, **11**, and **13** displayed significant inhibition of collagen accumulation with weak cytotoxicity in HFL1 cells. Trichocarboline A (**1**) stood out to be the most active compound for further examination, inhibiting collagen accumulation to 85.21 ± 3.2% at 10 μM. Although pirfenidone exhibited a slightly higher inhibition rate compared to **1**, it exerted more cytotoxicity effects on HFL1 cells, which was consistent with microscopic observations (Figure 3).

**TABLE 2:** Collagen accumulation inhibition rate (IR) and cell survival rate (SR) of **1**–**3**, **5**–**25**.

Compounds	Inhibition rate (%)	Survival rate (%)	Compounds	Inhibition rate (%)	Survival rate (%)
**1**	85.21 ± 3.15	80.01 ± 0.15	**15**	12.99 ± 7.07	96.64 ± 3.08
**2**	29.98 ± 2.04	96.84 ± 0.66	**16**	33.25 ± 1.50	93.68 ± 1.73
**3**	21.16 ± 1.50	80.76 ± 1.19	**17**	47.96 ± 2.47	98.19 ± 0.54
**5**	25.41 ± 0.57	86.47 ± 3.83	**18**	5.15 ± 3.53	87.37 ± 3.54
**6**	8.42 ± 0.57	92.83 ± 3.78	**19**	1.55 ± 1.50	94.09 ± 1.94
**7**	27.04 ± 3.71	91.33 ± 6.49	**20**	37.83 ± 1.50	91.13 ± 0.66
**8**	43.38 ± 1.96	84.37 ± 2.60	**21**	5.47 ± 3.44	86.12 ± 2.59
**9**	21.16 ± 2.71	93.69 ± 0.52	**22**	1.88 ± 1.13	87.08 ± 3.83
**10**	26.39 ± 5.74	94.79 ± 2.62	**23**	5.47 ± 3.00	93.84 ± 3.86
**11**	62.66 ± 2.04	96.29 ± 3.35	**24**	47.63 ± 4.63	89.88 ± 3.46
**12**	35.21 ± 3.00	87.38 ± 0.84	**25**	3.51 ± 2.47	85.52 ± 1.14
**13**	73.77 ± 3.40	91.89 ± 0.45	**pirfenidone**	87.83 ± 8.34	69.64 ± 0.80
**14**	36.85 ± 1.50	97.54 ± 1.52			

### Trichocarboline A **(1)** Suppressed the Expressions of Fibrotic Biomarkers

**FIGURE 4:**
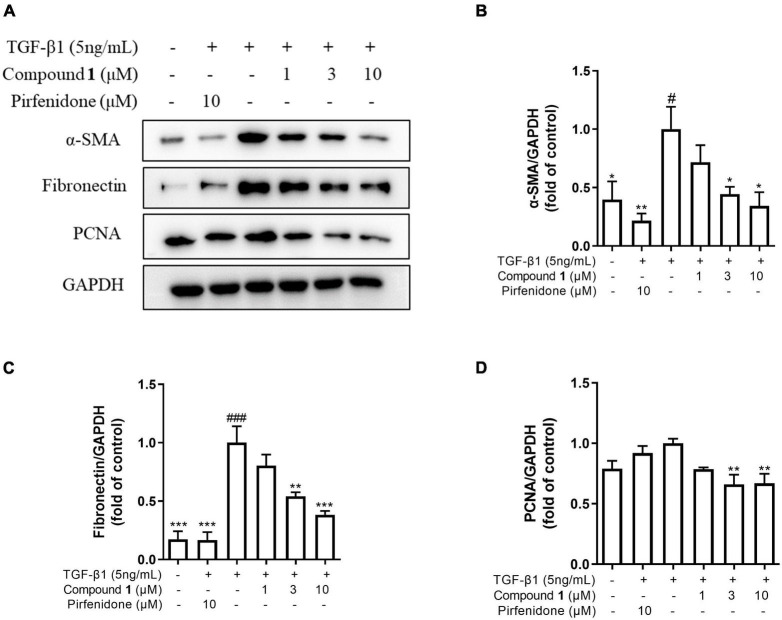
Trichocarboline A (**1**) inhibited extracellular matrix (ECM) deposition induced by transforming growth factor (TGF)-β1 in HFL1 cells. **(A–D)** HFL1 cells were treated with various concentrations of compound **1** (0, 1, 3, 10 μM) or pirfenidone in the presence or absence of TGF-β1 (5 ng/mL) stimulation for 48 h. The protein expression of **(B)** alpha smooth muscle actin (α-SMA), **(C)** fibronectin (FN), and **(D)** proliferating cell nuclear antigen (PCNA) was analyzed by Western blot. *n* = 3. Data were presented as the mean ± SEM. *P < 0.05, **P < 0.01, ***P < 0.001 vs. the TGF-β1 group. ^#^*P* < 0.05, ^##^*P* < 0.01, ^###^*P* < 0.001 vs. the control group.

To investigate the mechanism of the anti-fibrotic activity of trichocarboline A (**1**), it was evaluated for its ability to inhibit TGF-β1-induced fibronectin (FN) and α-smooth muscle actin (α-SMA) expression in HFL1 cells. FN and α-SMA have been commonly considered fibrotic markers, as they are overexpressed in fibrotic diseases. TGF-β1 can also upregulate the expression of proliferating cell nuclear antigen (PCNA), which is a component of the replication and repair machinery (Kelman, 1997). Therefore, the ability of trichocarboline A (**1**) to inhibit the expression of PCNA was also evaluated. Trichocarboline A (**1**) also reduced the TGF-β1-induced PCNA protein level in a dose-dependent manner, indicating that trichocarboline A (**1**) can inhibit the excessive proliferation of cells. As shown in Figure 4, trichocarboline A (**1**) reduced TGF-β1-induced FN and α-SMA expression in HFL1 cells, which is consistent with its ability to reduce ECM deposition, suggesting that trichocarboline A (**1**) was a potential anti-fibrotic agent.

### Trichocarboline A **(1)** Inhibited Extracellular Matrix Deposition *via* Inhibition of TGF-β/Smad Signaling

**FIGURE 5:**
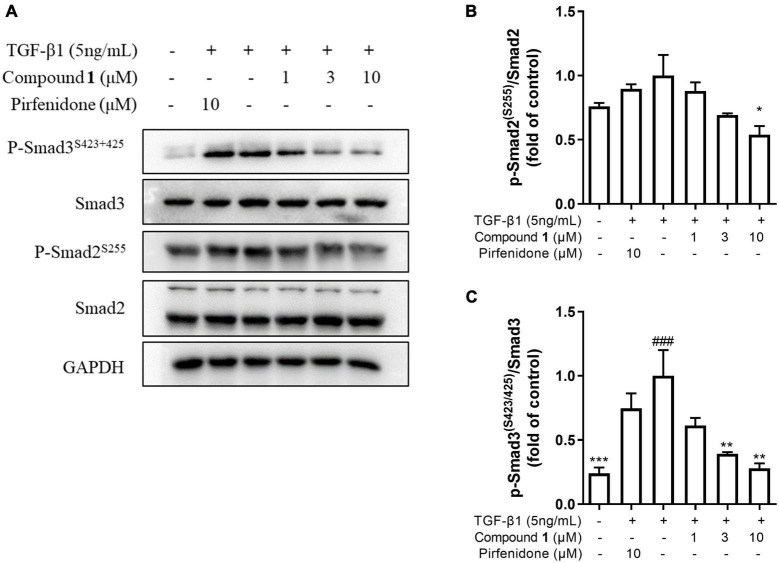
Effect of trichocarboline A (**1**) on Smad signaling pathway. **(A–C)** HFL1 cells were treated with various concentrations of compound **1** (0, 1, 3, 10 μM) or pirfenidone in the presence or absence of transforming growth factor TGF-β1 (5 ng/mL) stimulation for 30 min. The protein expression of **(B)** p-Smad2^Ser255^, and **(C)** p-Smad3^Ser423/425^ was analyzed by Western blotting. *n* = 3. Data were presented as the mean ± SEM. *P < 0.05, **P < 0.01, ***P < 0.001 vs. the TGF-β1 group. ^#^*P* < 0.05, ^##^*P* < 0.01, ^###^*P* < 0.001 vs. the control group.

TGF-β/Smad signaling pathway mainly involves intracellular phosphorylation cascade of Smad-2/3 transcription factors. Phosphorylated Smad-2/3 complex with Smad-4, and translocate to the nucleus, then complex drive the expression of target matrix genes, finally activating the expressions of ECM proteins (Walton et al., 2017). To determine whether trichocarboline A (**1**) could inhibit this signaling pathway, the protein levels of phosphorylated Smad2 and Smad3 (p-Smad2/3) in TGF-β1-induced HFL1 cells were investigated. As anticipated, the expressions of p-Smad2 and p-Smad3 were markedly increased by TGF-β1 stimulation, whereas trichocarboline A (**1**) down-regulated their expressions in a dose-dependent manner (Figure 5). During this process, the total expressions of Smad2 and Smad3 had no significant changes. These evidences suggested that trichocarboline A (**1**) suppressed the phosphorylation Smad2/3 in TGF-β/Smad signaling.

**FIGURE 6:**
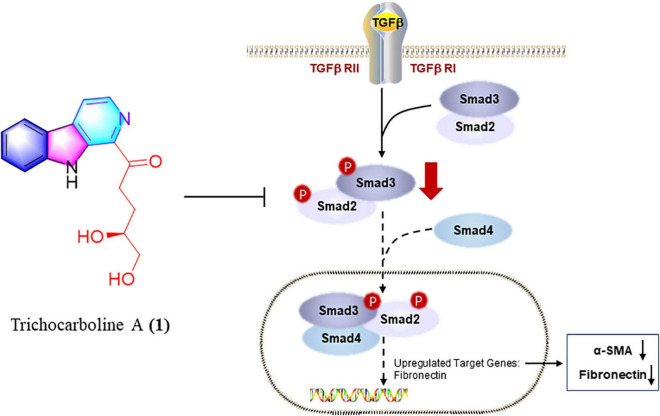
Trichocarboline A (**1**) suppressed the phosphorylation of Smad2/3 in TGF-β/Smad signaling. Trichocarboline A (**1**) was a TGF-β/Smad signaling inhibitor, making it a promising lead compound for the development of agents to treat pulmonary fibrosis disease.

## Discussion

Lung damage caused by pulmonary fibrosis cannot be repaired, and current options for drugs and therapies are limited. For critically ill patients, lung transplantation is the only option. Two drugs, nintedanib and pirfenidone, are currently on the market for the prevention of mild pulmonary fibrosis. A review concluded that pirfenidone appears to improve progression-free survival in patients with idiopathic pulmonary fibrosis, but has a lesser effect on lung function (Spagnolo et al., 2010). Nintedanib has been shown to slow the decline in forced vital capacity, but not improve survival in patients with fibrosis (Dimitroulis, 2014). Discovery of new anti-pulmonary fibrosis therapies remains a key challenge.

*Trichoderma* species have been demonstrated as a promising source of secondary metabolites with significant bioactivities, including antimicrobial sesquiterpenes, antioxidant mycotoxin, antibiotic peptaibols, antiviral trichokonins, and cytotoxic terpenes (Li M.-F. et al., 2019). However, to the best of our knowledge, there have been no reports on the secondary metabolites from the genus *Trichoderma* as anti-pulmonary fibrosis agents. This study lays the foundation for the use of β-carbolines in the treatment of pulmonary fibrosis.

In summary, chemical investigations of the deep-sea fungus *Trichoderma* sp. MCCC 3A01244 led to the isolation of 25 compounds, including two new β-carbolines, trichocarbolines A and C (**1** and **4**). Trichocarboline B [(+)- and (–)-enantiomers] are reported for the first time as naturally occurring metabolites. Compounds **1**, **11**, and **13** showed inhibitory activity against collagen accumulation in HFL1 cells. Furthermore, trichocarboline A (**1**) can suppress the expression of FN, α-SMA, and PCNA in TGF-β1-induced HFL1 cells, and reduce ECM deposition. Trichocarboline A (**1**) down-regulated phosphorylating Smad 2 and Smad 3. Thus, trichocarboline A (**1**) may reduce the accumulation of heteromeric Smad complex (phosphorylating Smad 2 and Smad 3 and binding to Smad 4) in the nucleus, thereby down-regulating the transcription of fibrosis genes, including α-SMA and fibronectin, which is expected further study. Mechanistic study revealed that trichocarboline A (**1**) was a TGF-β/Smad signaling inhibitor, making it a promising lead compound for the development of drugs to treat pulmonary fibrosis disease.

## Data Availability Statement

The original contributions presented in this study are included in the article/Supplementary Material, further inquiries can be directed to the corresponding author.

## Author Contributions

W-JL conceived and designed the study and finalized the manuscript. M-JH and P-NC carried out the experiments. M-JH wrote the manuscript. W-JL, H-JL, FW, G-YZ, Z-ZS, X-PL, W-ZM, and JX guided experiments. TM revised the manuscript. All authors provided critical feedback and helped shape the research, analysis, and manuscript and generated in-house and no manuscript mill was used, and agreed to be accountable for all aspects of work ensuring integrity and accuracy.

## Conflict of Interest

The authors declare that the research was conducted in the absence of any commercial or financial relationships that could be construed as a potential conflict of interest.

## Publisher’s Note

All claims expressed in this article are solely those of the authors and do not necessarily represent those of their affiliated organizations, or those of the publisher, the editors and the reviewers. Any product that may be evaluated in this article, or claim that may be made by its manufacturer, is not guaranteed or endorsed by the publisher.
