# Supplementary material for: β-Carboline Alkaloids From the Deep-Sea Fungus Trichoderma sp. MCCC 3A01244 as a New Type of Anti-pulmonary Fibrosis Agent That Inhibits TGF-β/Smad Signaling Pathway
Source: Front Microbiol. 2022 Jul 28;13:947226. doi: 10.3389/fmicb.2022.947226 (PMC9366743; doi:10.3389/fmicb.2022.947226)

***Supplementary Material***

β-Carboline alkaloids from the deep-sea fungus *Trichoderma* sp. MCCC 3A01244 as a new type of anti-pulmonary fibrosis agent that inhibits TGF-β/Smad signaling pathway

Meng-Jiao Hao, Pei-Nan Chen, Hou-Jin Li, Feng Wu, Guang-Yu Zhang, Zong-Ze Shao, Xiu-Pian Liu, Wen-Zhe Ma, Jun Xu, Taifo Mahmud, and Wen-Jian Lan^*^

School of Pharmaceutical Sciences, Sun Yat-Sen University, Guangzhou 510006,

Guangdong, China

**Correspondence**

Wen-Jian Lan, School of Pharmaceutical Sciences, Sun Yat-Sen University, 132 East Circle Road at University City, Guangzhou 510006, China.

E-mail: lanwj@mail.sysu.edu.cn (W.-J. Lan).

## Table of Contents

[Supplementary Figure 1 HR-ESI-MS spectrum of trichocarboline A (**1**) 5](#_Toc76910291)

[Supplementary Figure 2 ^1^H NMR spectrum of trichocarboline A (**1**) in methanol-*d*_4_ (400 MHz) 6](#_Toc76910292)

[Supplementary Figure 3 ^13^C NMR spectrum of trichocarboline A (**1**) in methanol-*d*_4_ (100 MHz) 6](#_Toc76910293)

[Supplementary Figure 4 DEPT 135 spectrum of trichocarboline A (**1**) in methanol-*d*_4_ (100 MHz) 7](#_Toc76910294)

[Supplementary Figure 5 HSQC spectrum of trichocarboline A (**1**) in methanol-*d*_4_ 7](#_Toc76910295)

[Supplementary Figure 6 ^1^H-^1^H COSY spectrum of trichocarboline A (**1**) in methanol-*d*_4_ 8](#_Toc76910296)

Supplementary Figure 7 HMBC spectrum of trichocarboline A (**1**) in methanol-*d*_4_ 9

[Supplementary Figure 8 HR-ESI-MS spectrum of trichocarboline B (**2**) 9](#_Toc76910298)

Supplementary Figure 9 ^1^H NMR spectrum of trichocarboline B (**2**) in methanol-*d*_4_ (400 MHz) 11

Supplementary Figure 10 ^13^C NMR spectrum of trichocarboline B (**2**) in methanol-*d*_4_ (100 MHz) 11

Supplementary Figure 11 DEPT 135 spectrum of trichocarboline B (**2**) in methanol-*d*_4_ (100 MHz) 12

Supplementary Figure 12 HSQC spectrum of trichocarboline B (**2**) in methanol-*d*_4_ 13

Supplementary Figure 13 ^1^H-^1^H COSY spectrum of trichocarboline B (**2**) in methanol-*d*_4_ 13

Supplementary Figure 14 HMBC spectrum of trichocarboline B (**2**) in methanol-*d*_4_ 14

[Supplementary Figure 15 HR-ESI-MS spectrum of trichocarboline C (**4**) 15](#_Toc76910305)

[Supplementary Figure 16 ^1^H NMR spectrum of trichocarboline C (**4**) in CDCl_3_ (400 MHz) 16](#_Toc76910306)

[Supplementary Figure 17 ^13^C NMR spectrum of trichocarboline C (**4**) in CDCl_3_ (100 MHz) 17](#_Toc76910307)

[Supplementary Figure 18 DEPT 135 spectra of trichocarboline C (**4**) 18](#_Toc76910308)

[Supplementary Figure 19 HMQC spectrum of trichocarboline C (**4**) in CDCl_3_ 19](#_Toc76910309)

[Supplementary Figure 20 HMBC spectrum of trichocarboline C (**4**) in CDCl_3_ 20](#_Toc76910310)

[Supplementary Figure 21 COSY spectrum of trichocarboline C (4) in CDCl_3_ 21](#_Toc76910311)

[Supplementary Figure 22 ^1^H NMR spectrum of **5** in acetone-*d*_6_ (400 MHz) 22](#_Toc76910312)

[Supplementary Figure 23 ^13^C NMR spectrum of **5** in acetone-*d*_6_ (100 MHz) 23](#_Toc76910313)

[Supplementary Figure 24 ^1^H NMR spectrum of **6** in acetone-*d*_6_ (400 MHz) 24](#_Toc76910314)

[Supplementary Figure 25 ^13^C NMR spectrum of **6** in acetone-*d*_6_ (100 MHz) 24](#_Toc76910315)

[Supplementary Figure 26 ^1^H NMR spectrum of **7** in DMSO-*d*_6_ (400 MHz) 26](#_Toc76910316)

[Supplementary Figure 27 ^13^C NMR spectrum of **7** in DMSO-*d*_6_ (100 MHz) 26](#_Toc76910317)

[Supplementary Figure 28 ^1^H NMR spectrum of **8** in acetone-*d*_6_ (400 MHz) 27](#_Toc76910318)

[Supplementary Figure 29 ^13^C NMR spectrum of **8** in acetone-*d*_6_ (100 MHz) 27](#_Toc76910319)

[Supplementary Figure 30 ^1^H NMR spectrum of **9** in methanol-*d*_4_ (400 MHz) 28](#_Toc76910320)

[Supplementary Figure 31 ^13^C NMR spectrum of **9** in methanol-*d*_4_ (100 MHz) 28](#_Toc76910321)

[Supplementary Figure 32 ^1^H NMR spectrum of **10** in DMSO-*d*_6_ (400 MHz) 29](#_Toc76910322)

[Supplementary Figure 33 ^13^C NMR spectrum of **10** in DMSO-*d*_6_ (100 MHz) 29](#_Toc76910323)

[Supplementary Figure 34 ^1^H NMR spectrum of **11** in DMSO-*d*_6_ (400 MHz) 30](#_Toc76910324)

[Supplementary Figure 35 ^13^C NMR spectrum of **11** in DMSO-*d*_6_ (100 MHz) 30](#_Toc76910325)

[Supplementary Figure 36 ^1^H NMR spectrum of **12** in acetone-*d*_6_ (400 MHz) 31](#_Toc76910326)

[Supplementary Figure 37 ^13^C NMR spectrum of **12** in acetone-*d*_6_ (100 MHz) 31](#_Toc76910327)

[Supplementary Figure 38 ^1^H NMR spectrum of **13** in acetone-*d*_6_ (400 MHz) 32](#_Toc76910328)

[Supplementary Figure 39 ^13^C NMR spectrum of **13** in acetone-*d*_6_ (100 MHz) 32](#_Toc76910329)

[Supplementary Figure 40 ^1^H NMR spectrum of **14** in CDCl_3_ (400 MHz) 33](#_Toc76910330)

[Supplementary Figure 41 ^13^C NMR spectrum of **14** in CDCl_3_ (100 MHz) 33](#_Toc76910331)

[Supplementary Figure 42 ^1^H NMR spectrum of **15** in CDCl_3_ (400 MHz) 34](#_Toc76910332)

[Supplementary Figure 43 ^13^C NMR spectrum of **15** in CDCl_3_ (100 MHz) 34](#_Toc76910333)

[Supplementary Figure 44 ^1^H NMR spectrum of **16** in acetone-*d*_6_ (400 MHz) 35](#_Toc76910334)

[Supplementary Figure 45 ^13^C NMR spectrum of **16** in acetone-*d*_6_ (100 MHz) 35](#_Toc76910335)

[Supplementary Figure 46 ^1^H NMR spectrum of **17** in acetone-*d*_6_ (400 MHz) 36](#_Toc76910336)

[Supplementary Figure 47 ^13^C NMR spectrum of **17** in acetone-*d*_6_ (100 MHz) 36](#_Toc76910337)

[Supplementary Figure 48 ^1^H NMR spectrum of **18** in methanol-*d*_4_ (400 MHz) 37](#_Toc76910338)

[Supplementary Figure 49 ^13^C NMR spectrum of **18** in methanol-*d*_4_ (100 MHz) 37](#_Toc76910339)

[Supplementary Figure 50 ^1^H NMR spectrum of **19** in acetone-*d*_6_ (400 MHz) 38](#_Toc76910340)

[Supplementary Figure 51 ^13^C NMR spectrum of **19** in acetone-*d*_6_ (100 MHz) 38](#_Toc76910341)

[Supplementary Figure 52 ^1^H NMR spectrum of **20** in acetone-*d*_6_ (400 MHz) 39](#_Toc76910342)

[Supplementary Figure 53 ^13^C NMR spectrum of **20** in acetone-*d*_6_ (100MHz) 39](#_Toc76910343)

[Supplementary Figure 54 ^1^H NMR spectrum of **21** in acetone-*d*_6_ (400MHz) 40](#_Toc76910344)

[Supplementary Figure 55 ^13^C NMR spectrum of **21** in acetone-*d*_6_ (100 MHz) 40](#_Toc76910345)

[Supplementary Figure 56 ^1^H NMR spectrum of **22** in CDCl_3_ (400 MHz) 41](#_Toc76910346)

[Supplementary Figure 57 ^13^C NMR spectrum of **22** in CDCl_3_ (100 MHz) 41](#_Toc76910347)

[Supplementary Figure 58 ^1^H NMR spectrum of **23** in acetone-*d*_6_ (400 MHz) 42](#_Toc76910348)

[Supplementary Figure 59 ^13^C NMR spectrum of **23** in acetone-*d*_6_ (100 MHz) 42](#_Toc76910349)

[Supplementary Figure 60 ^1^H NMR spectrum of **24** in acetone-*d*_6_ (400 MHz) 43](#_Toc76910350)

[Supplementary Figure 61 ^13^C NMR spectrum of **24** in acetone-*d*_6_ (100 MHz) 43](#_Toc76910351)

[Supplementary Figure 62 ^1^H NMR spectrum of **25** in methanol-*d*_4_ (400 MHz) 44](#_Toc76910352)

[Supplementary Figure 63 ^13^C NMR spectrum of **25** in methanol-*d*_4_ (100 MHz) 44](#_Toc76910353)

#### **Supplementary Figure 1** HR-ESI-MS spectrum of trichocarboline A (**1**)


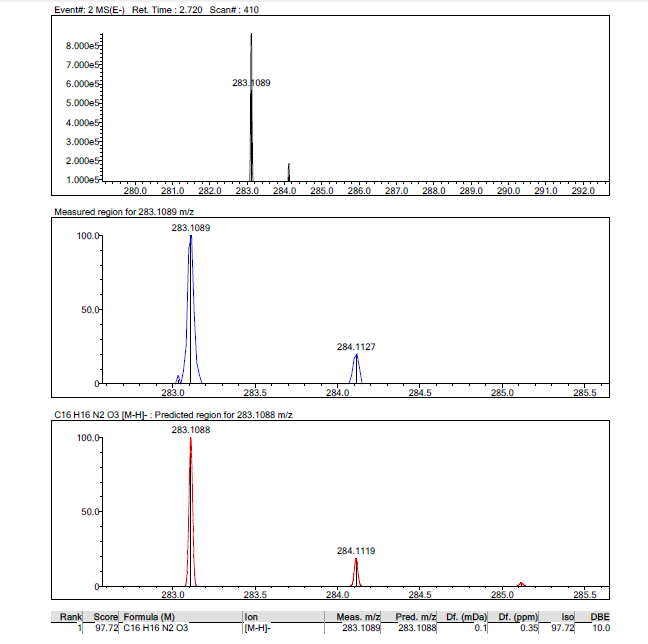


#### **Supplementary Figure 2** ^1^H NMR spectrum of trichocarboline A (**1**) in methanol-*d*_4_ (400MHz)


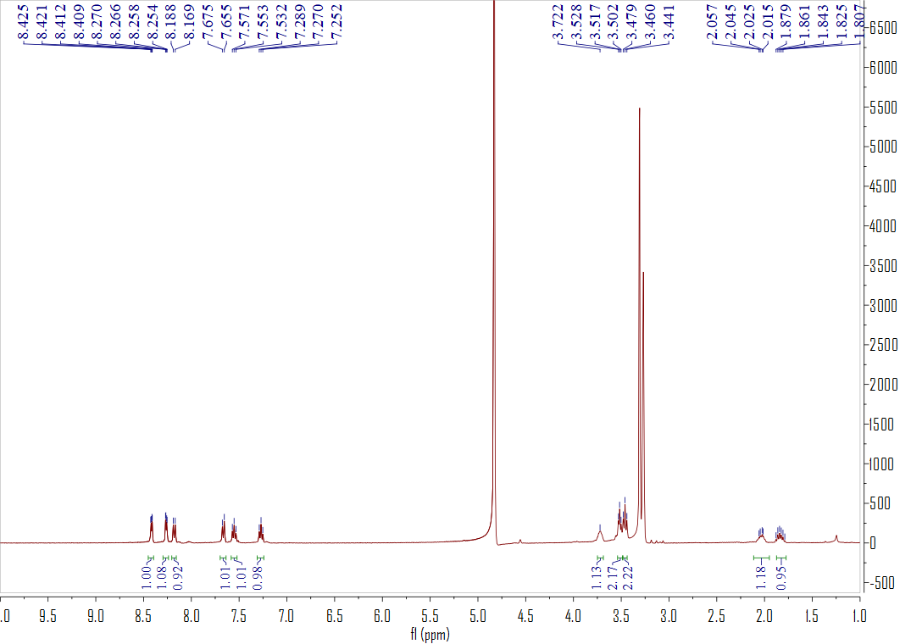


#### **Supplementary Figure 3** ^13^C NMR spectrum of trichocarboline A (**1**) in methanol-*d*_4_ (100MHz)


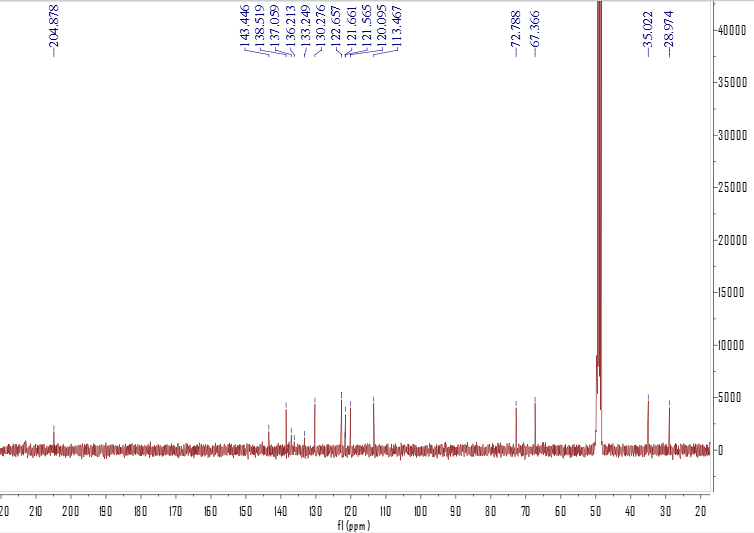


####

#### **Supplementary Figure 4** DEPT 135 spectrum of trichocarboline A (**1**) in methanol-*d*_4_ (100MHz)


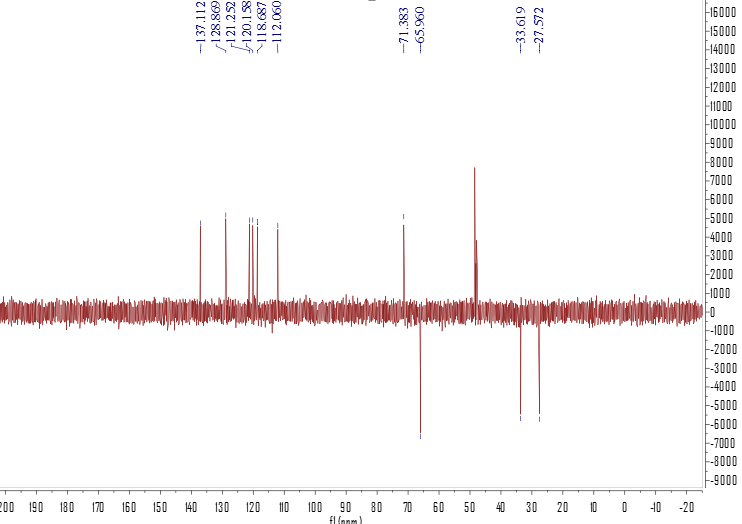


#### **Supplementary Figure 5** HSQC spectrum of trichocarboline A (**1**) in methanol-*d*_4_


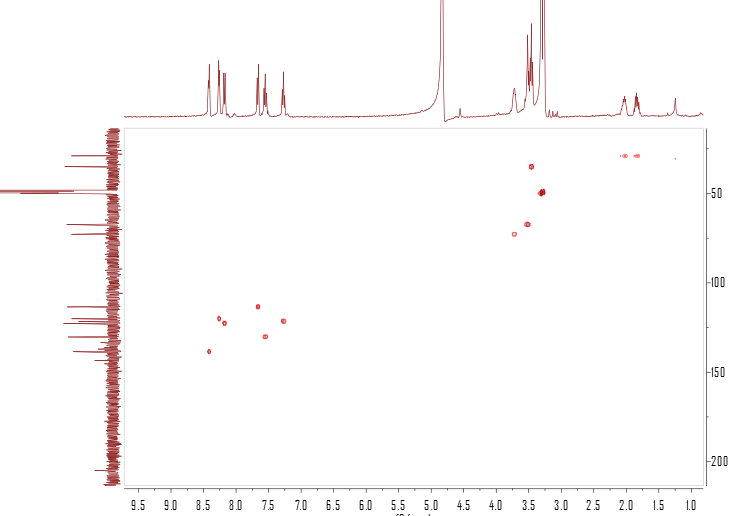


#### **Supplementary Figure 6** ^1^H-^1^H COSY spectrum of trichocarboline A (**1**) in methanol-*d*_4_


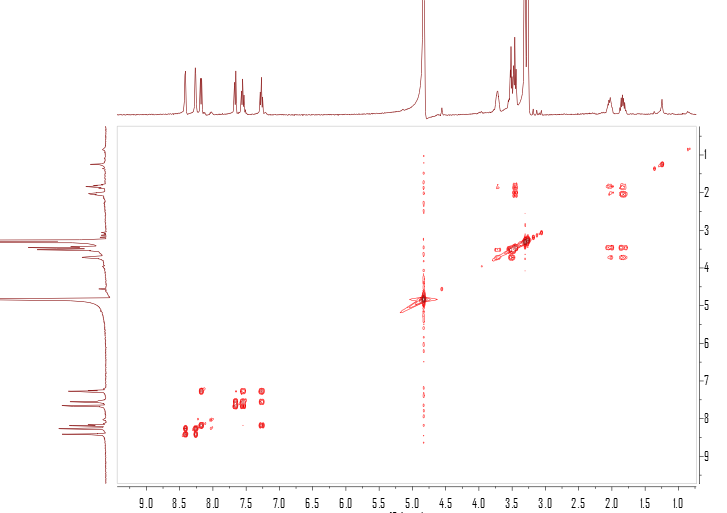


#### **Supplementary Figure 7** HMBC spectrum of trichocarboline A (**1**) in methanol-*d*_4_

**
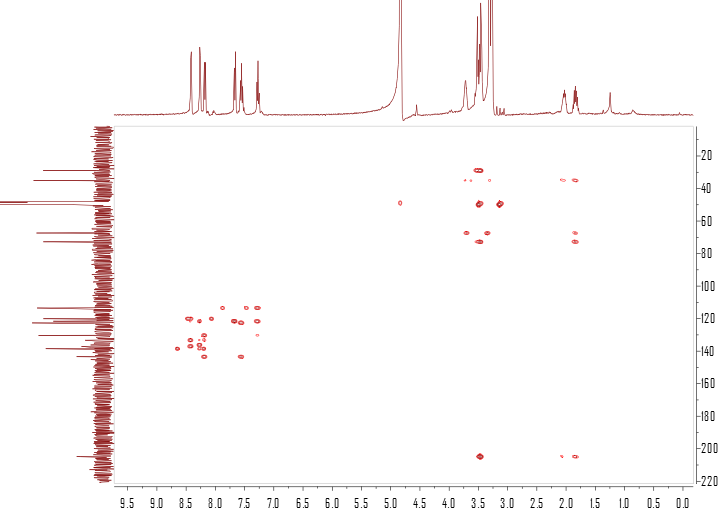
**

#### **Supplementary Figure 8** HR-ESI-MS spectrum of trichocarboline B (**2**)


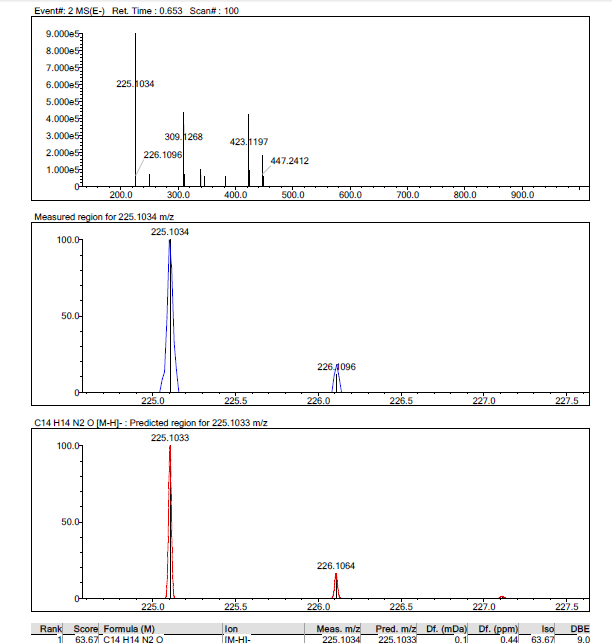


#### **Supplementary Figure 9** ^1^H NMR spectrum of trichocarboline B (**2**) in methanol-*d*_4_ (400MHz)


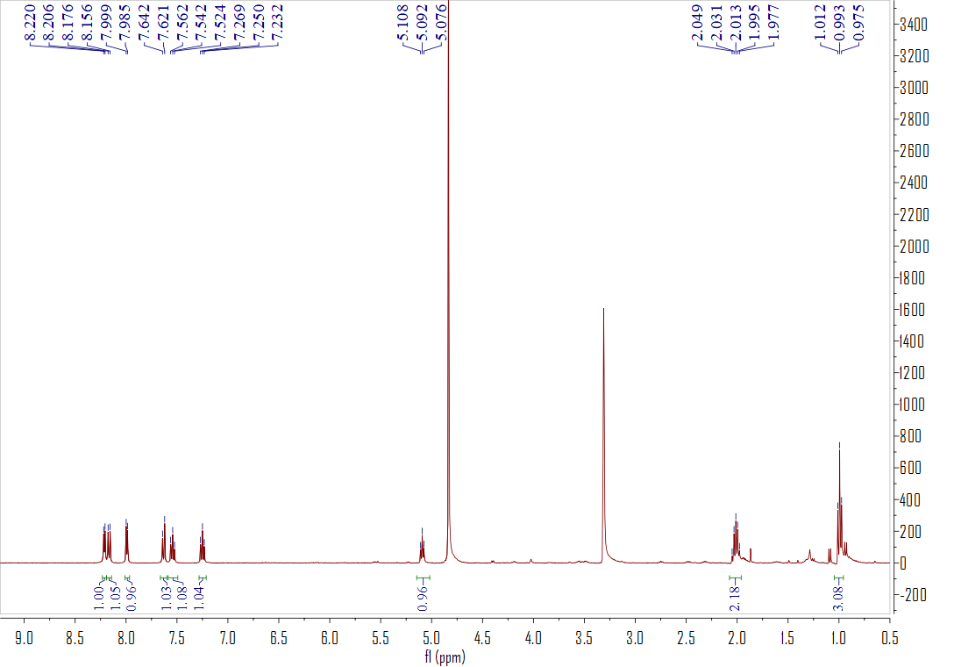


#### **Supplementary Figure 10** ^13^C NMR spectrum of trichocarboline B (**2**) in methanol-*d*_4_ (100MHz)


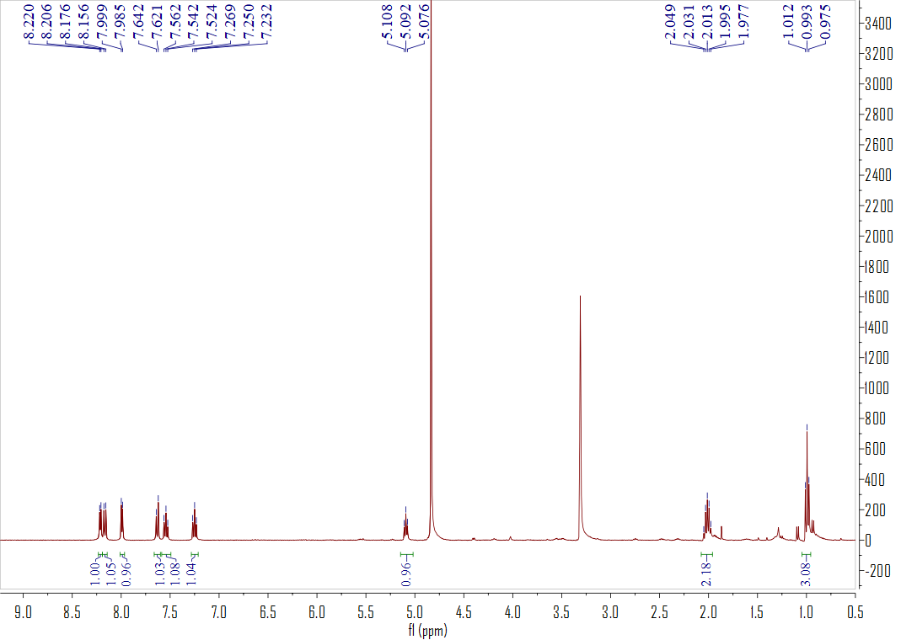


#### **Supplementary Figure 11** DEPT 135 spectrum of trichocarboline B (**2**) in methanol-*d*_4_ (100MHz)


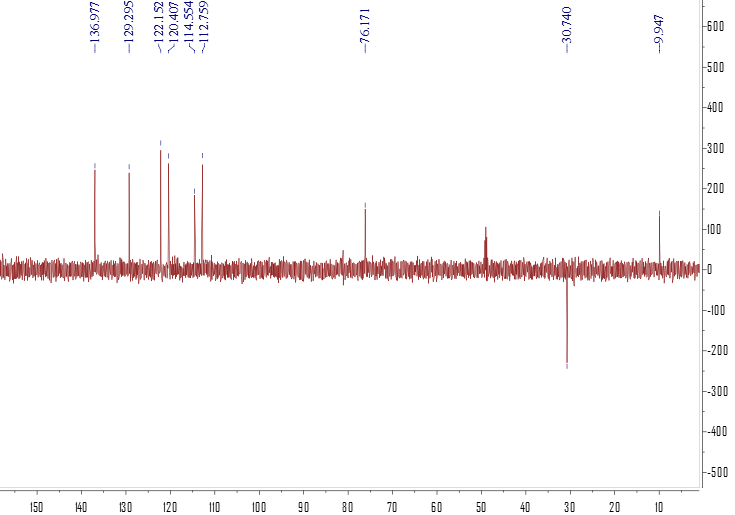


#### **Supplementary Figure 12** HSQC spectrum of trichocarboline B (**2**) in methanol-*d*_4_


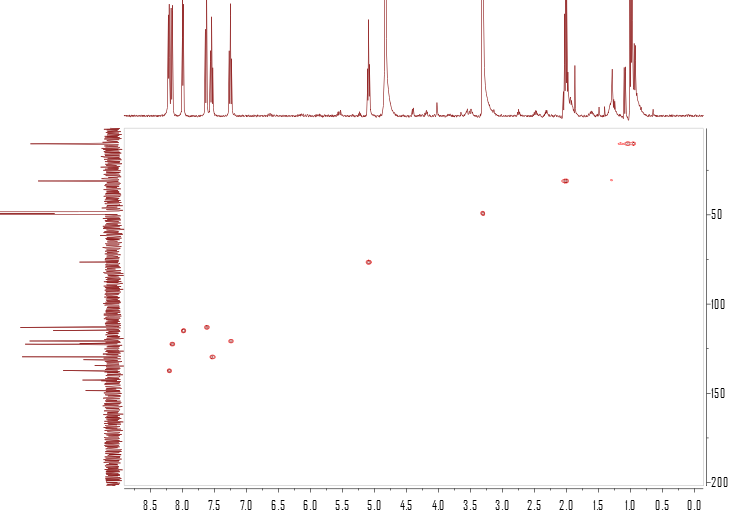


####

#### **Supplementary Figure 13** ^1^H-^1^H COSY spectrum of trichocarboline B (**2**) in methanol-*d*_4_


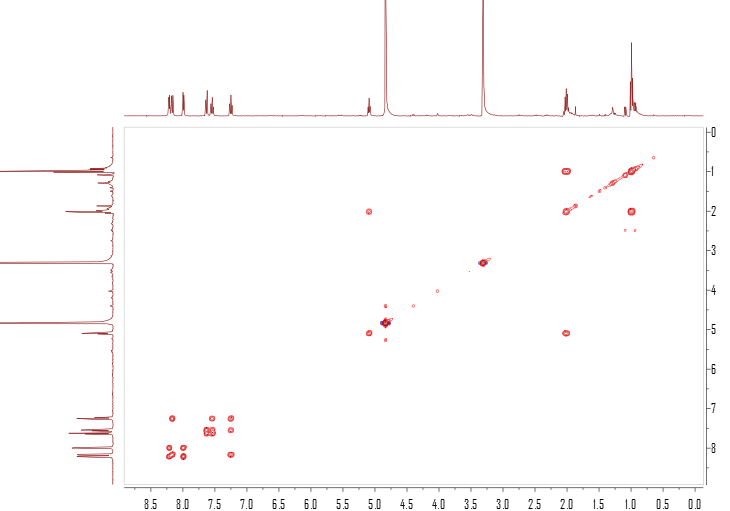


#### **Supplementary Figure 14** HMBC spectrum of trichocarboline B (**2**) in methanol-*d*_4_


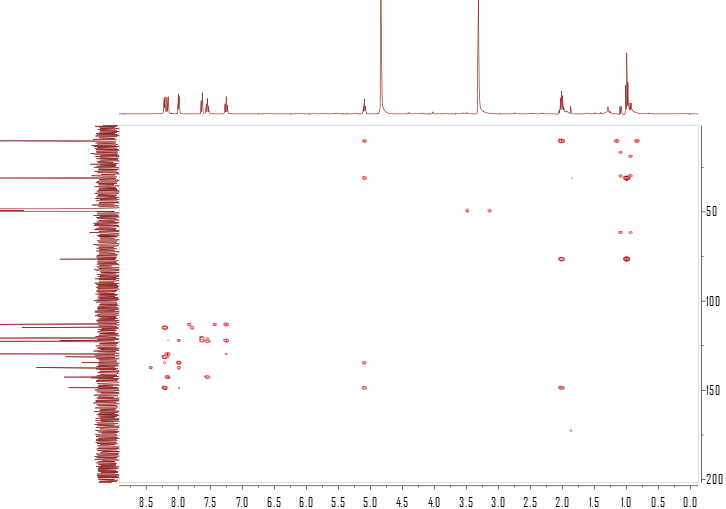


#### **Supplementary Figure 15** HR-ESI-MS spectrum of trichocarboline C (**4**)


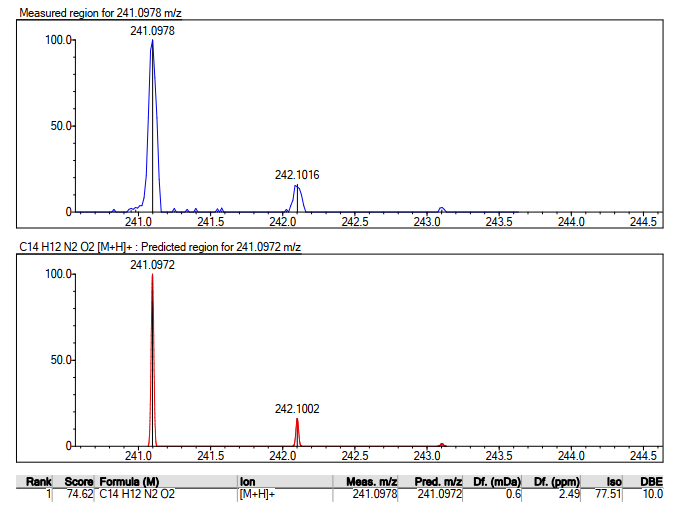


#### **Supplementary Figure 16** ^1^H NMR spectrum of trichocarboline C (**4**) in CDCl_3_ (400 MHz)


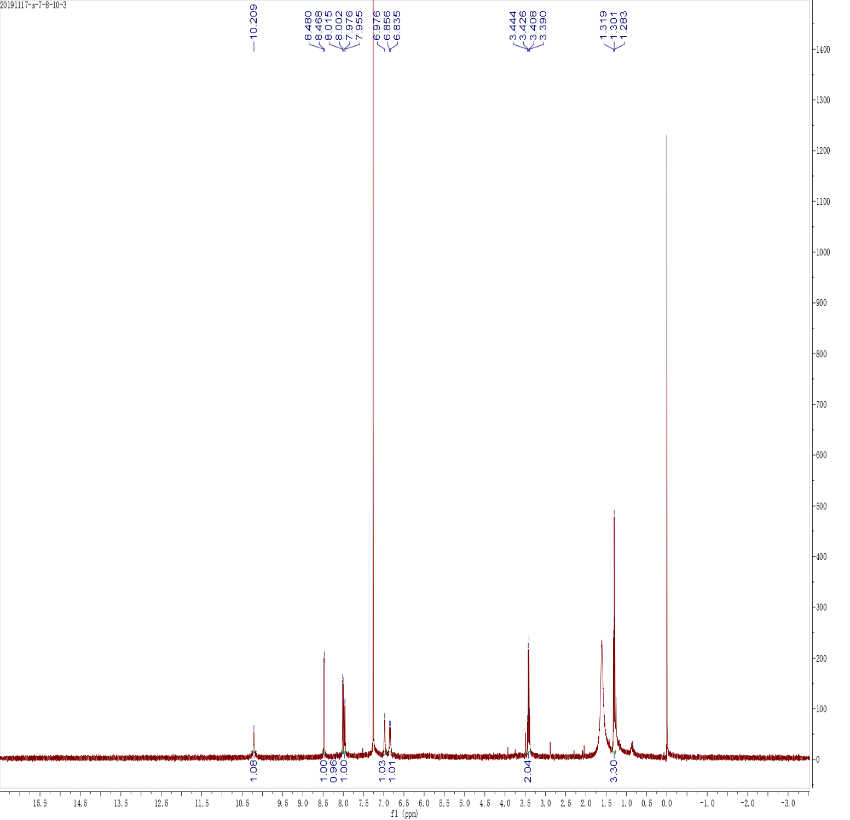


#### **Supplementary Figure 17** ^13^C NMR spectrum of trichocarboline C (**4**) in CDCl_3_ (100 MHz)


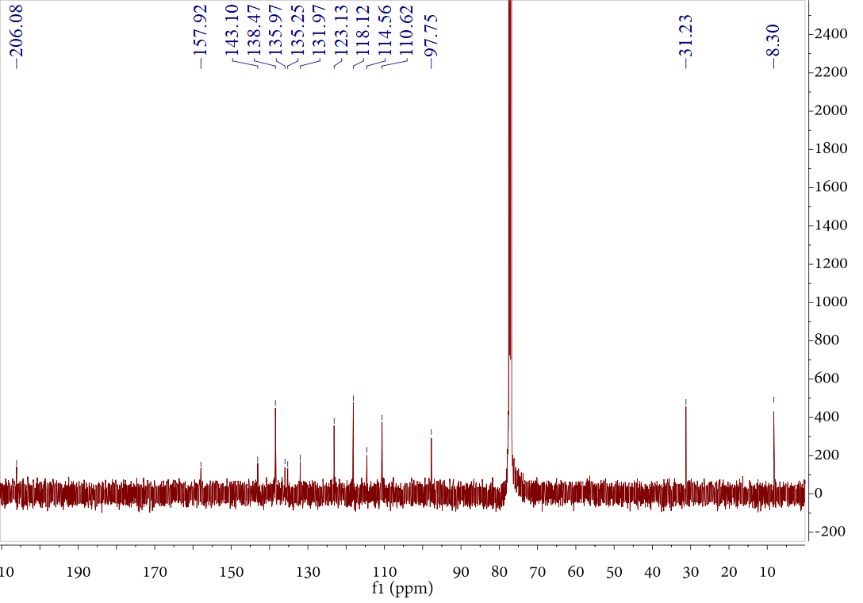


#### **Supplementary Figure 18** DEPT 135 spectra of trichocarboline C (**4**)


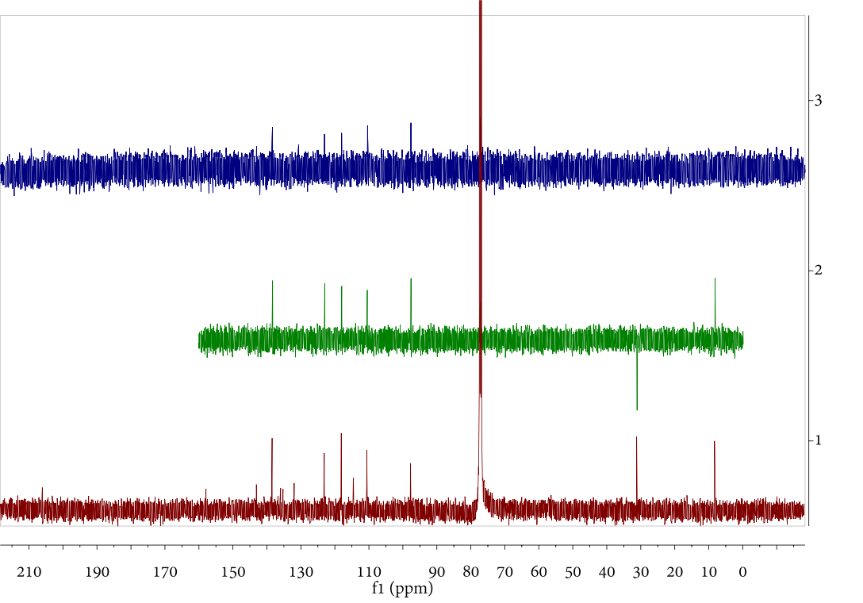


#### **Supplementary Figure 19** HSQC spectrum of trichocarboline C (**4**) in CDCl_3_


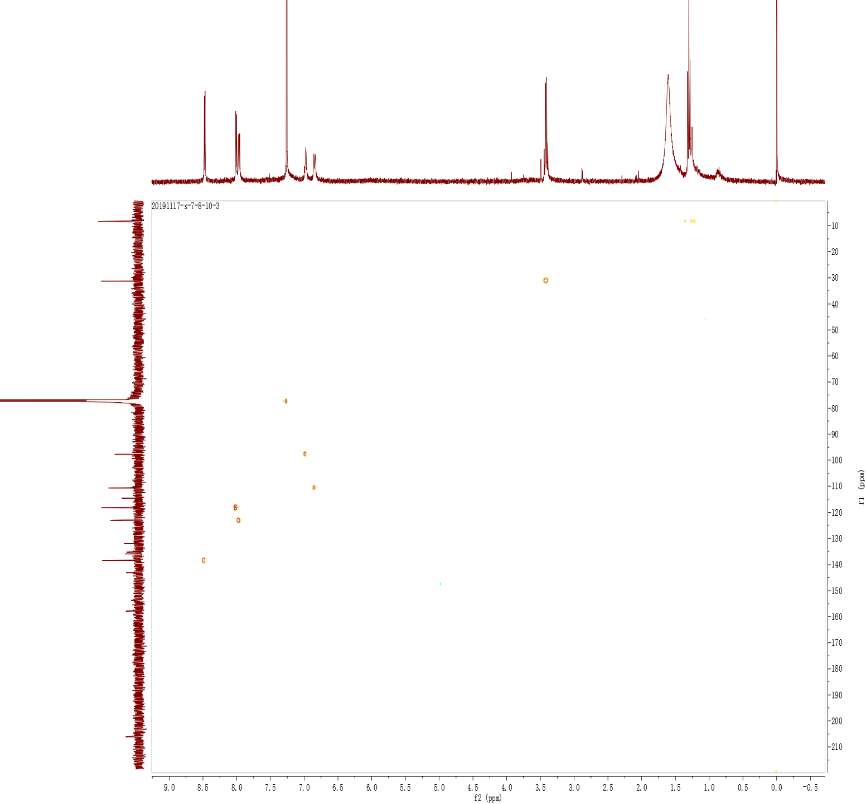


#### **Supplementary Figure 20** HMBC spectrum of trichocarboline C (**4**) in CDCl_3_


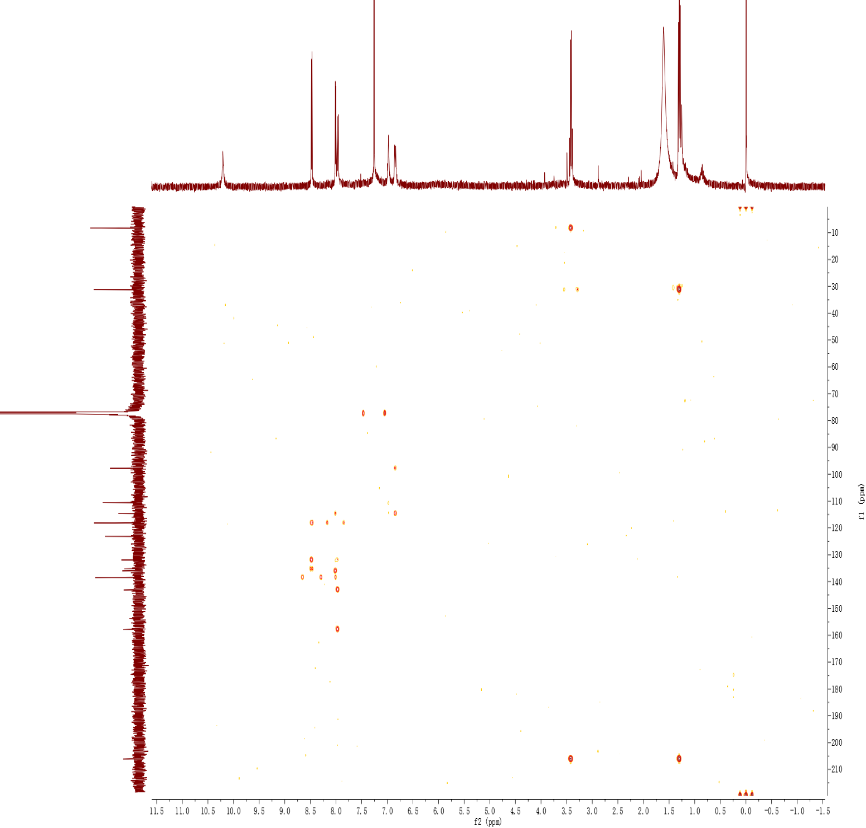


#### **Supplementary Figure 21** COSY spectrum of trichocarboline C (**4**) in CDCl_3_


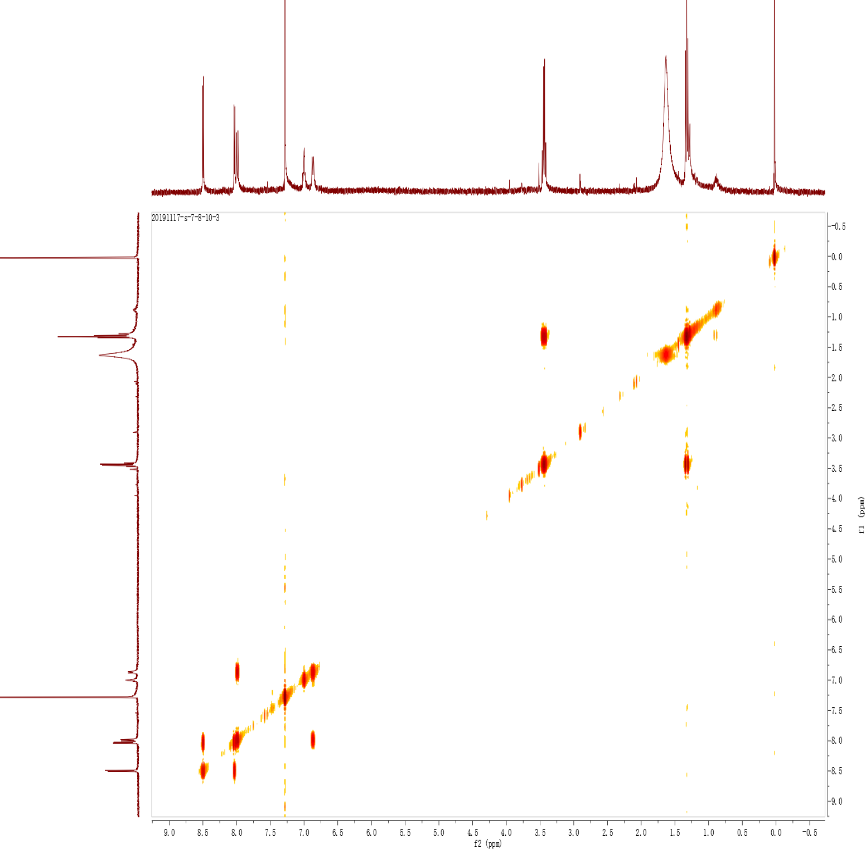


#### **Supplementary Figure 22** ^1^H NMR spectrum of **5** in acetone-*d*_6_ (400MHz)


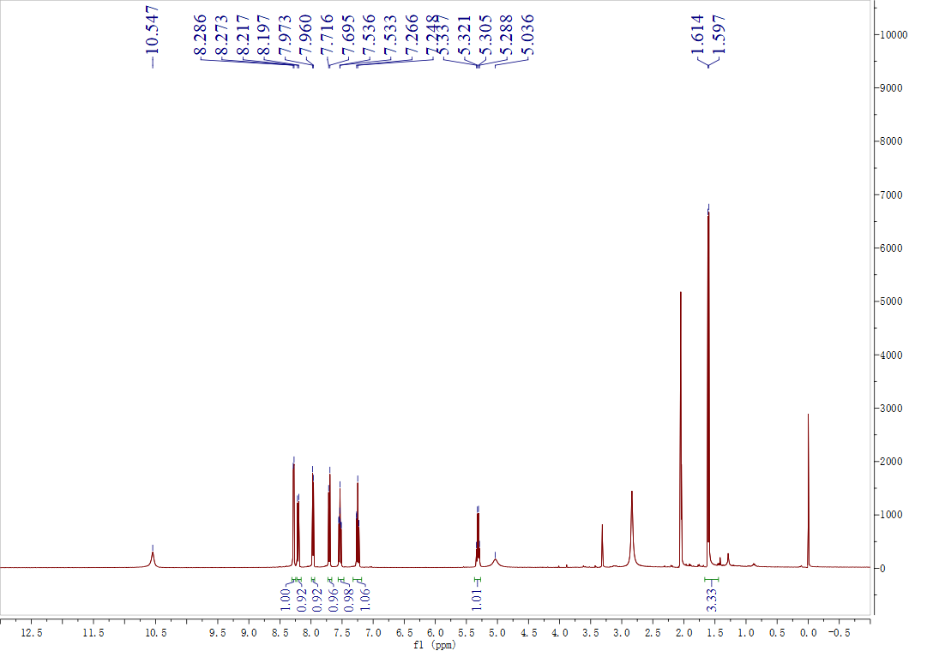


#### **Supplementary Figure 23** ^13^C NMR spectrum of **5** in acetone-*d*_6_ (100MHz)


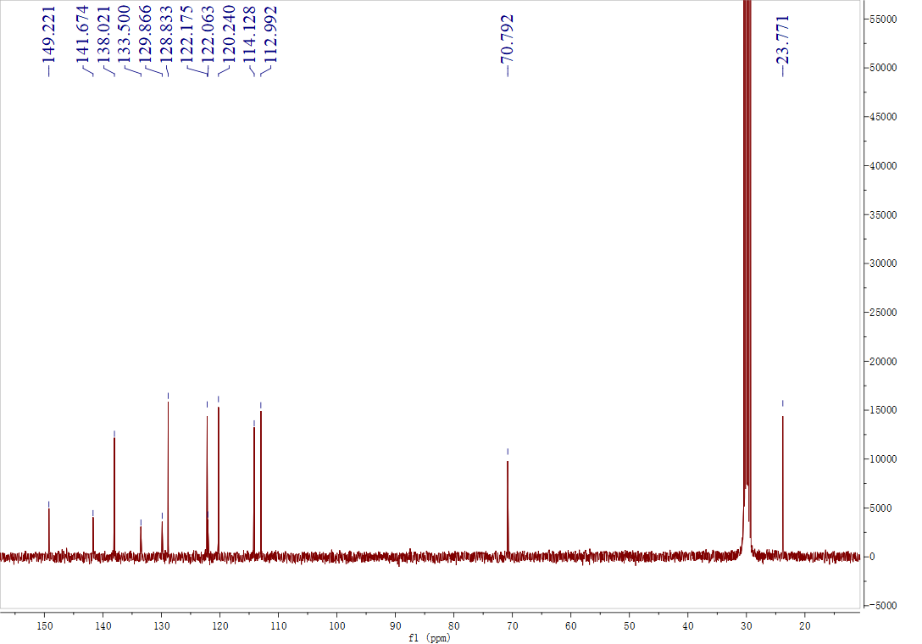


**Supplementary Figure 24** ^1^H NMR spectrum of **6** in acetone-*d*_6_ (400MHz)


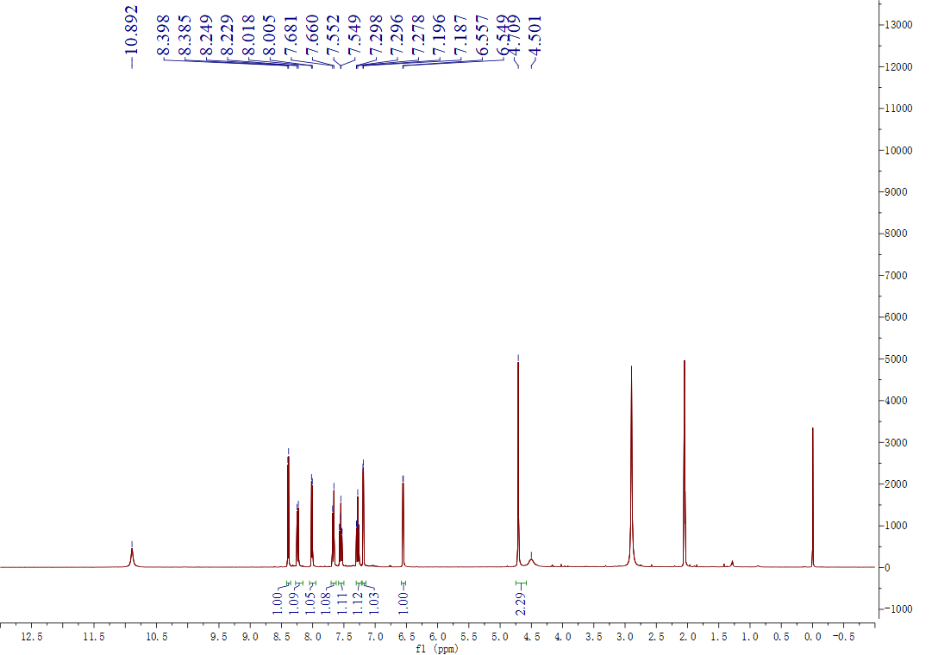


#### **Supplementary Figure 25** ^13^C NMR spectrum of **6** in acetone-*d*_6_ (100MHz)


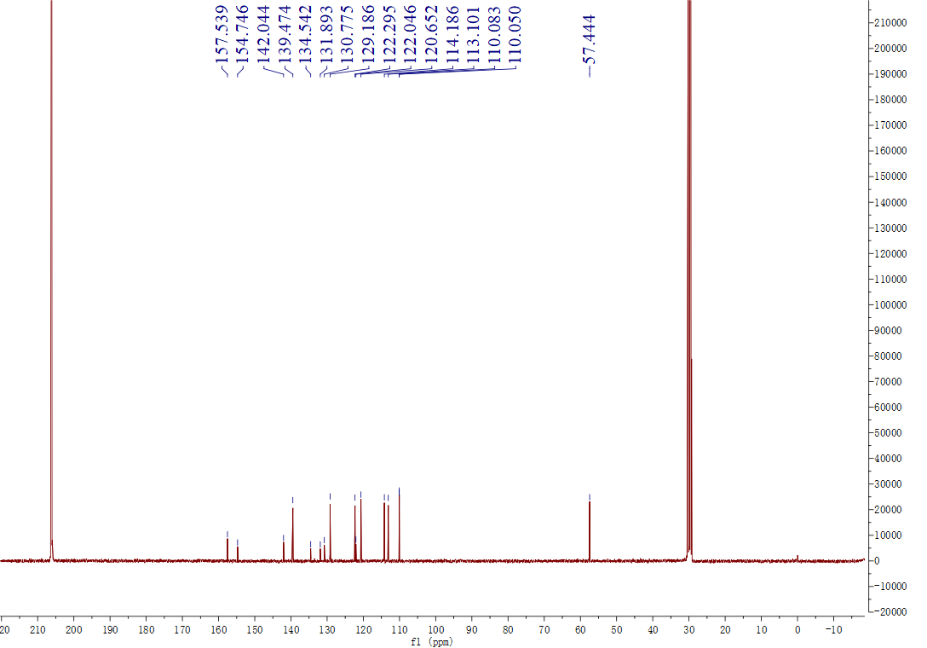


#### **Supplementary Figure 26** ^1^H NMR spectrum of **7** in DMSO-*d*_6_ (400MHz)


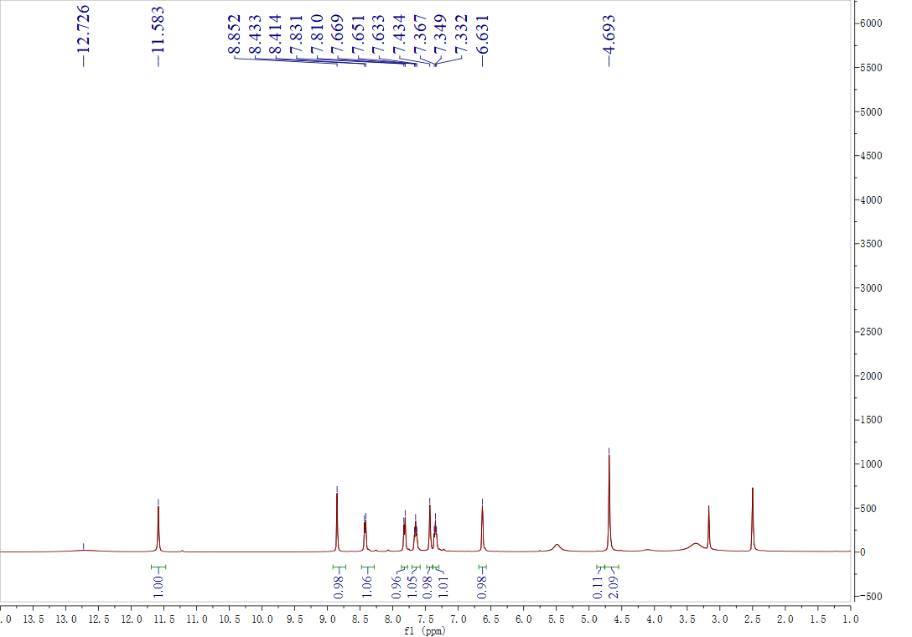


#### **Supplementary Figure 27** ^13^C NMR spectrum of **7** in DMSO-*d*_6_ (100MHz)


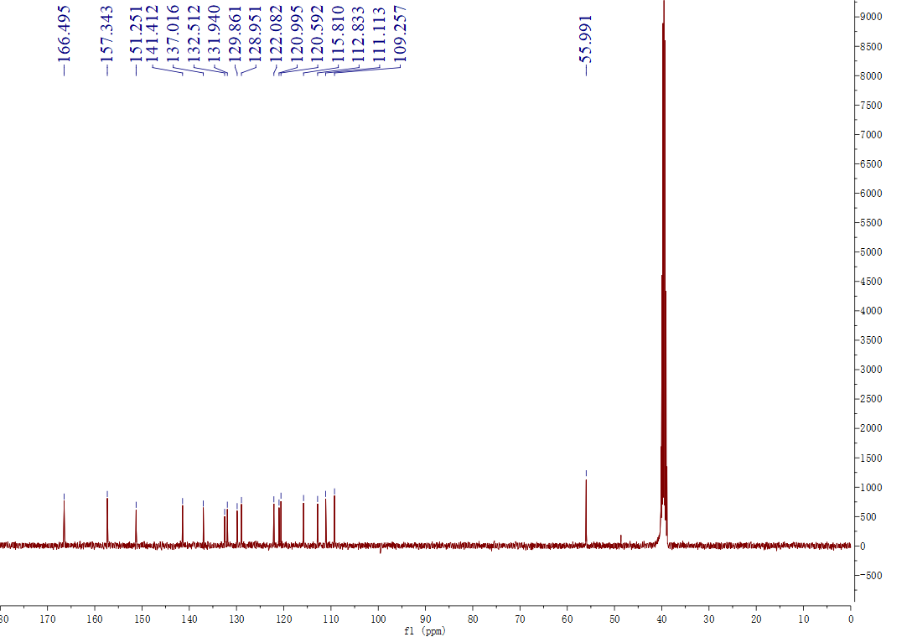


#### **Supplementary Figure 28** ^1^H NMR spectrum of **8** in acetone-*d*_6_ (400MHz)


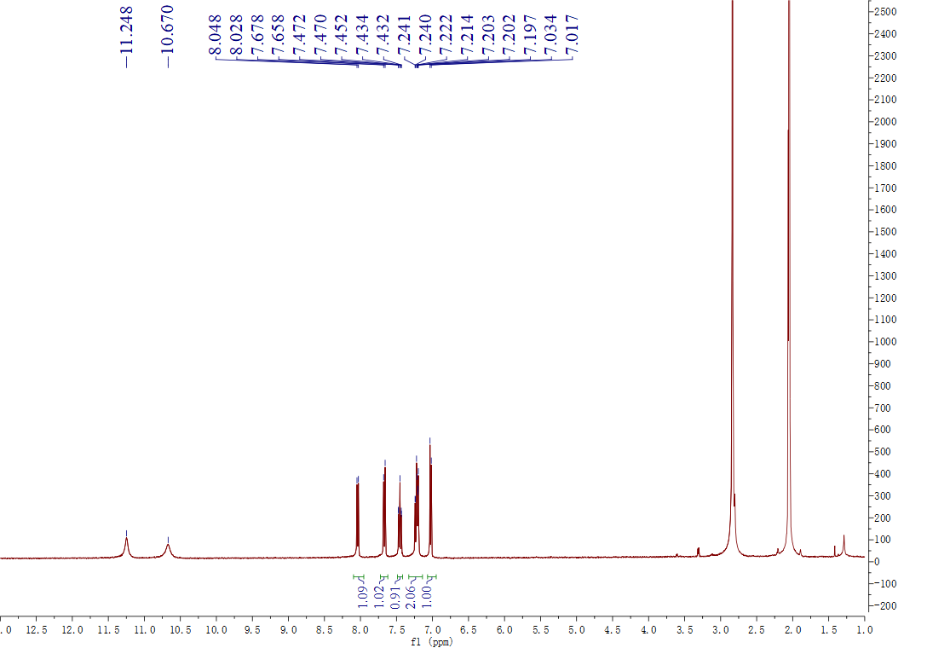


#### **Supplementary Figure 29** ^13^C NMR spectrum of **8** in acetone-*d*_6_ (100MHz)


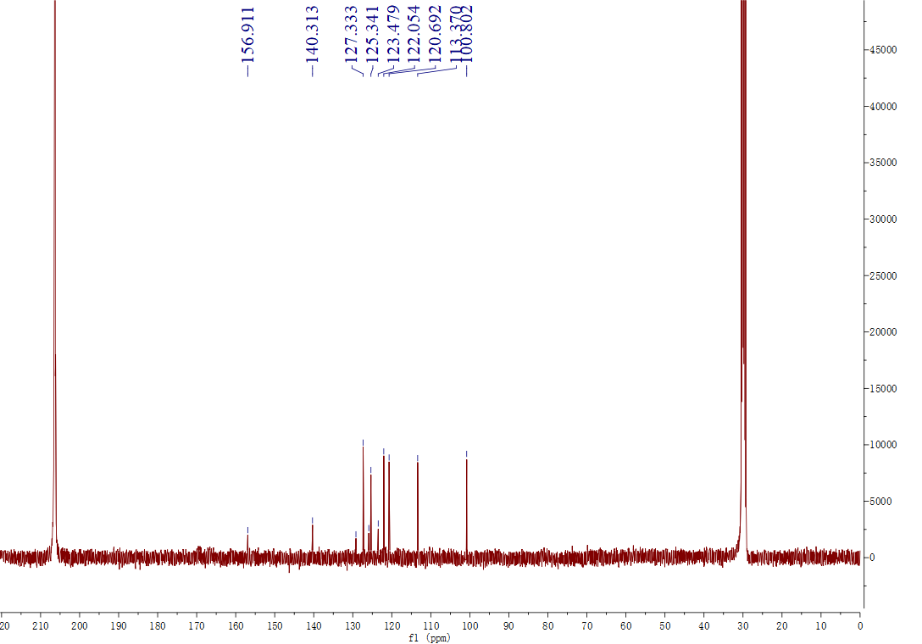


**Supplementary Figure 30** ^1^H NMR spectrum of **9** in methanol-*d*_4_ (400MHz)


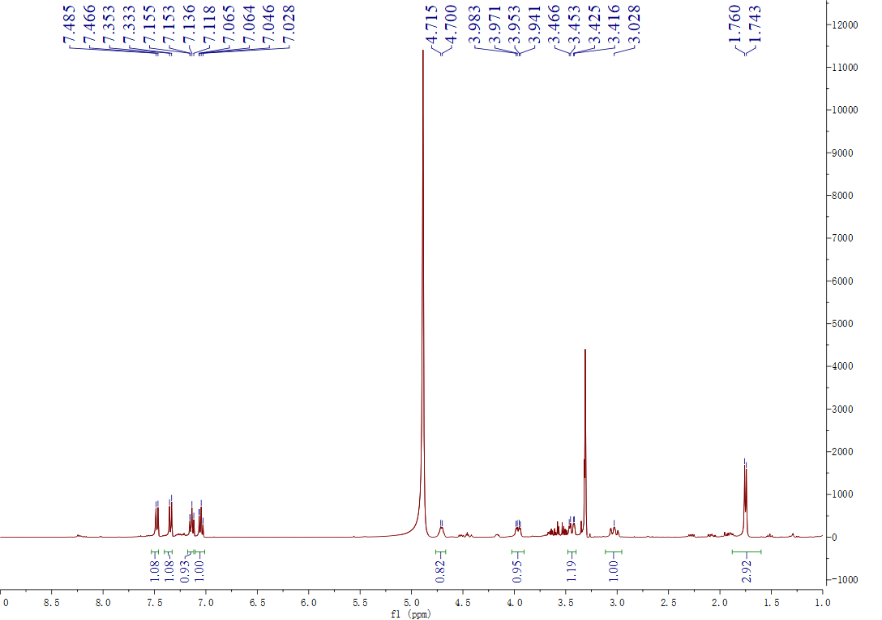


#### **Supplementary Figure 31** ^13^C NMR spectrum of **9** in methanol-*d*_4_ (100MHz)


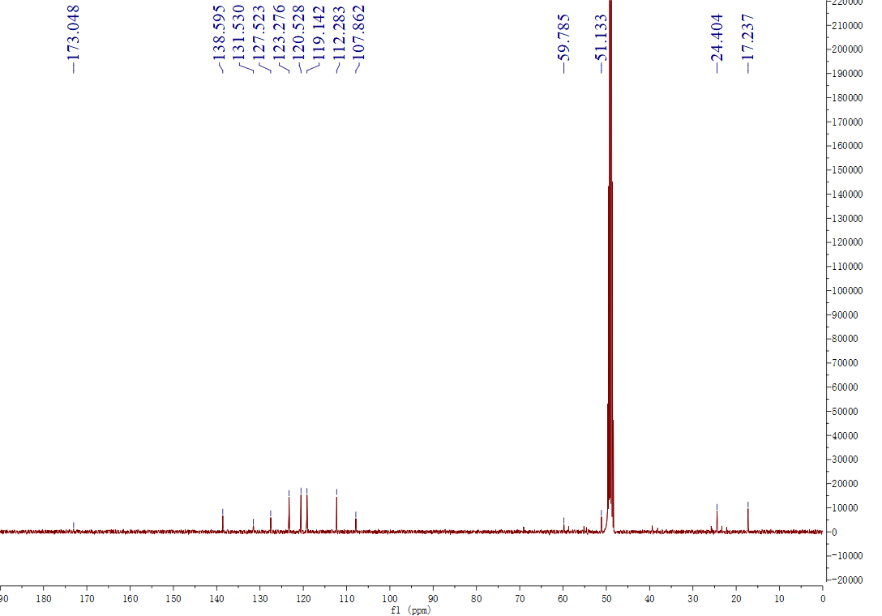


**Supplementary Figure 32** ^1^H NMR spectrum of **10** in DMSO-*d*_6_ (400MHz)


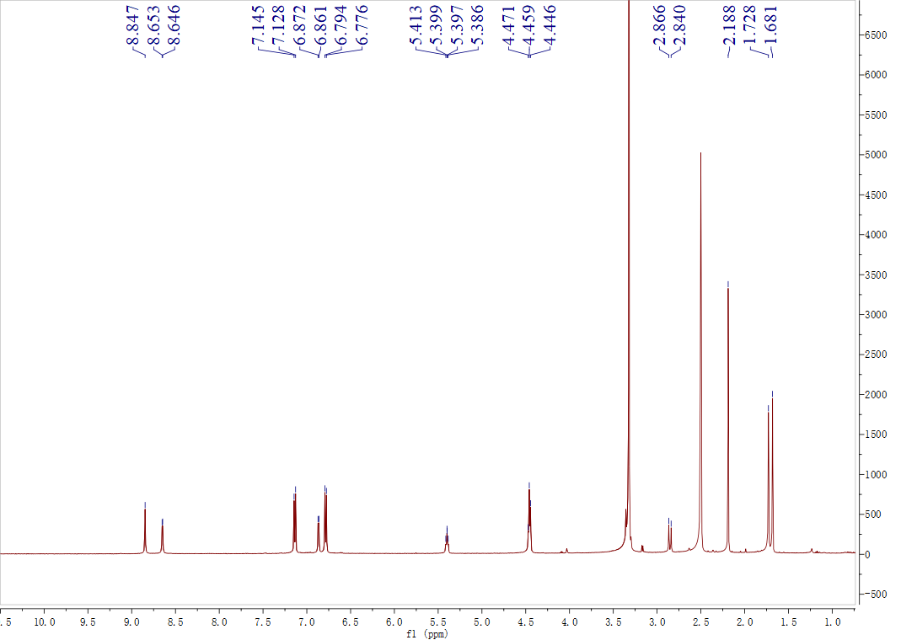


#### **Supplementary Figure 33** ^13^C NMR spectrum of **10** in DMSO-*d*_6_ (100MHz)


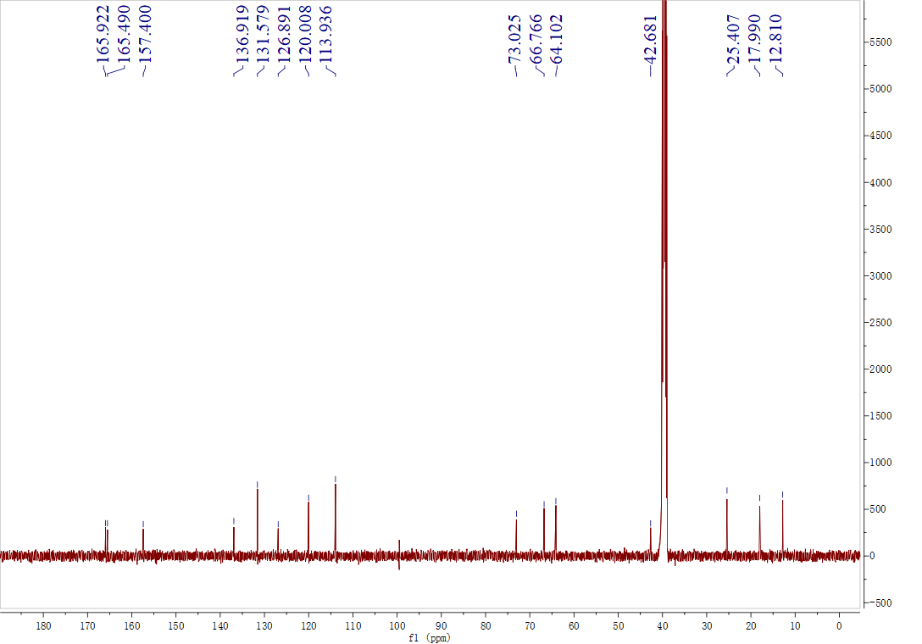


**Supplementary Figure 34** ^1^H NMR spectrum of **11** in DMSO-*d*_6_ (400MHz)


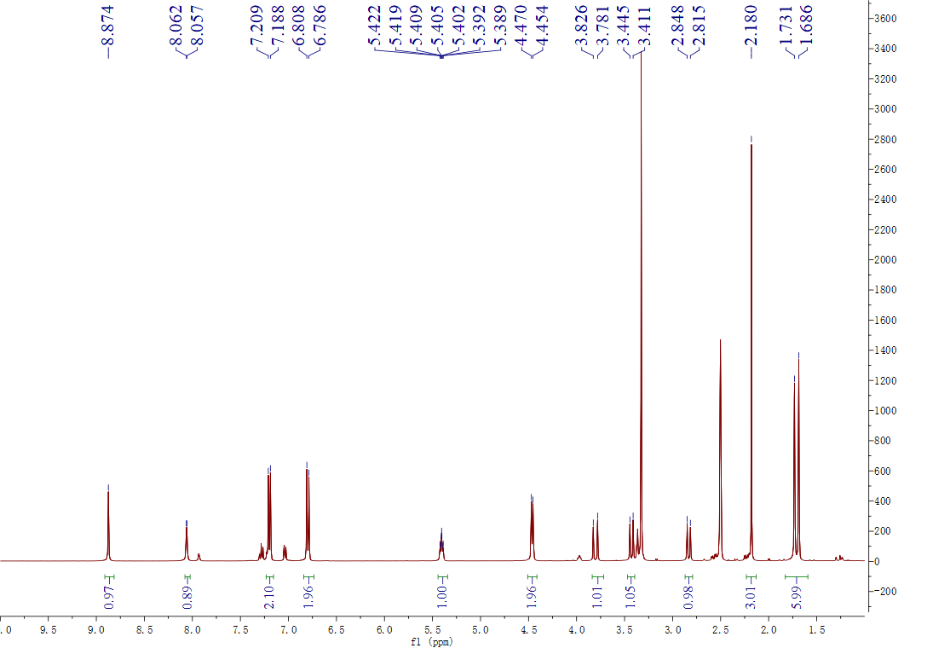


#### **Supplementary Figure 35** ^13^C NMR spectrum of **11** in DMSO-*d*_6_ (100MHz)


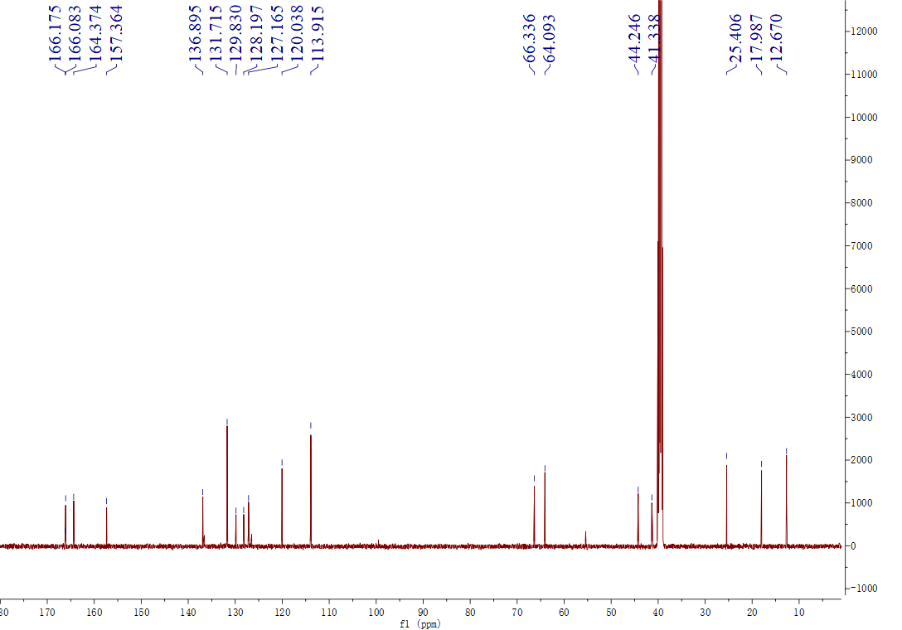


**Supplementary Figure 36** ^1^H NMR spectrum of **12** in acetone-*d*_6_ (400MHz)


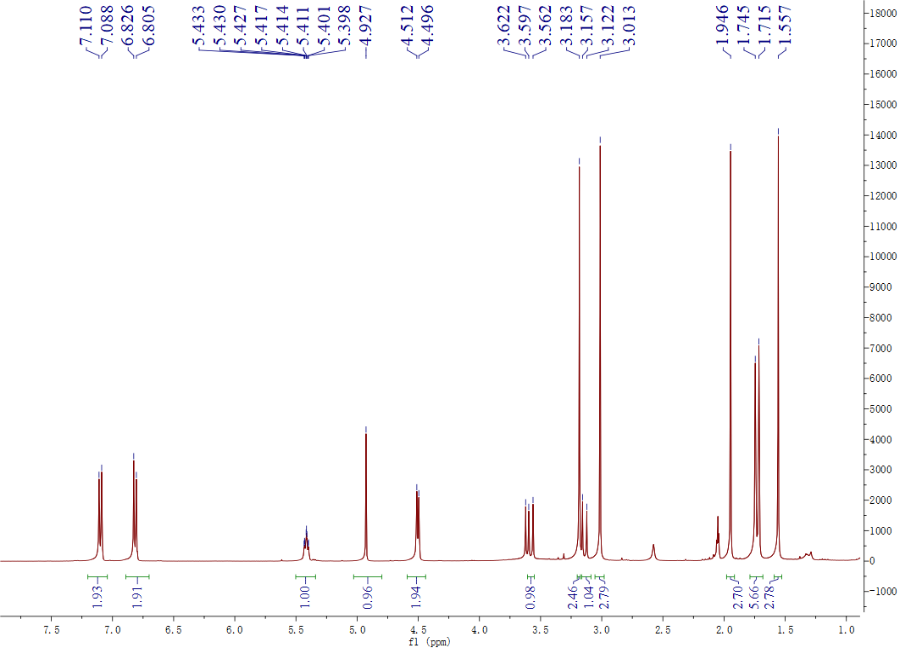


#### **Supplementary Figure 37** ^13^C NMR spectrum of **12** in acetone-*d*_6_ (100MHz)


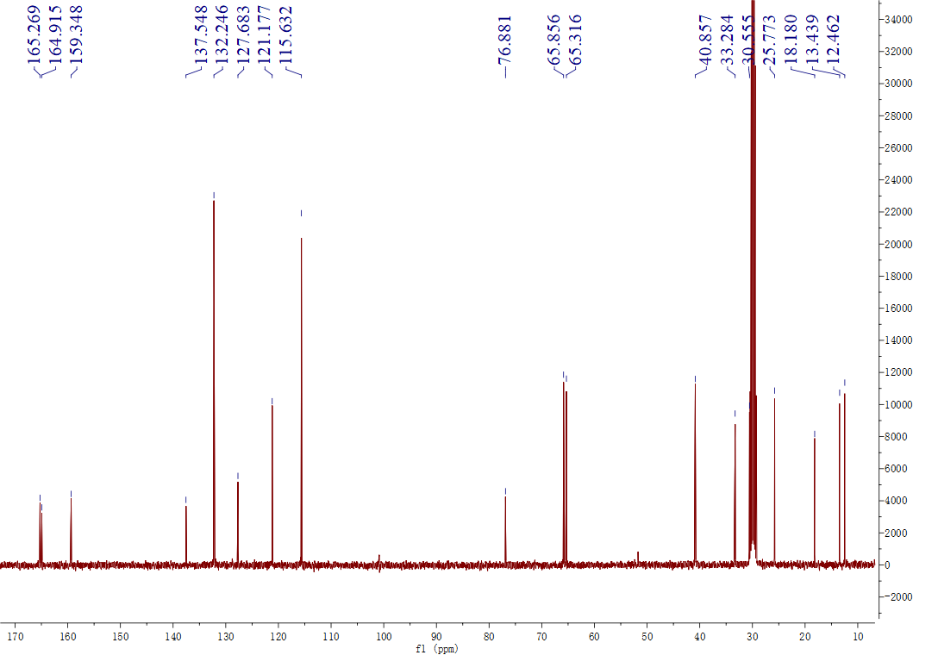


**Supplementary Figure 38** ^1^H NMR spectrum of **13** in acetone-*d*_6_ (400MHz)


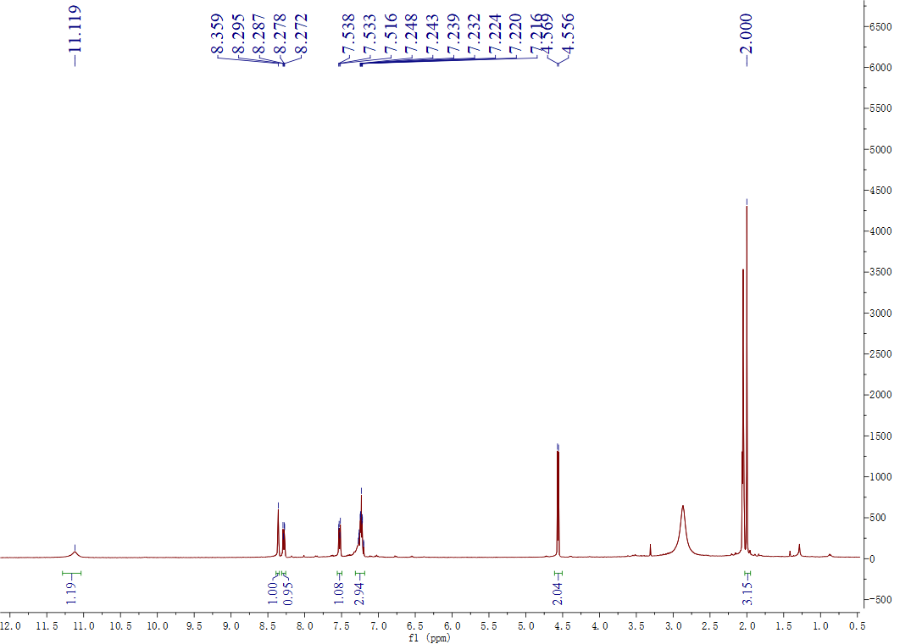


#### **Supplementary Figure 39** ^13^C NMR spectrum of **13** in acetone-*d*_6_ (100MHz)


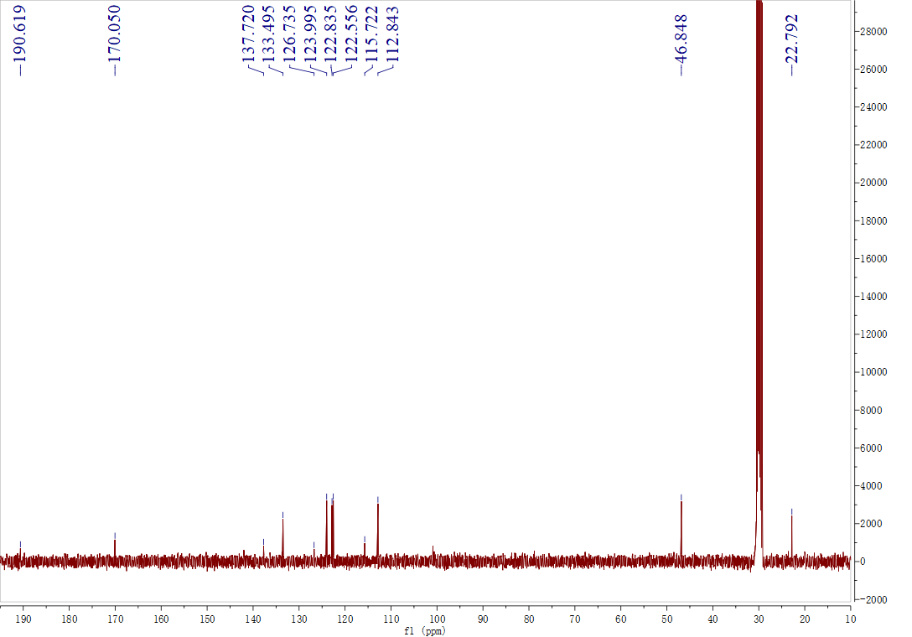


#### **Supplementary Figure 40** ^1^H NMR spectrum of **14** in CDCl_3_ (400MHz)


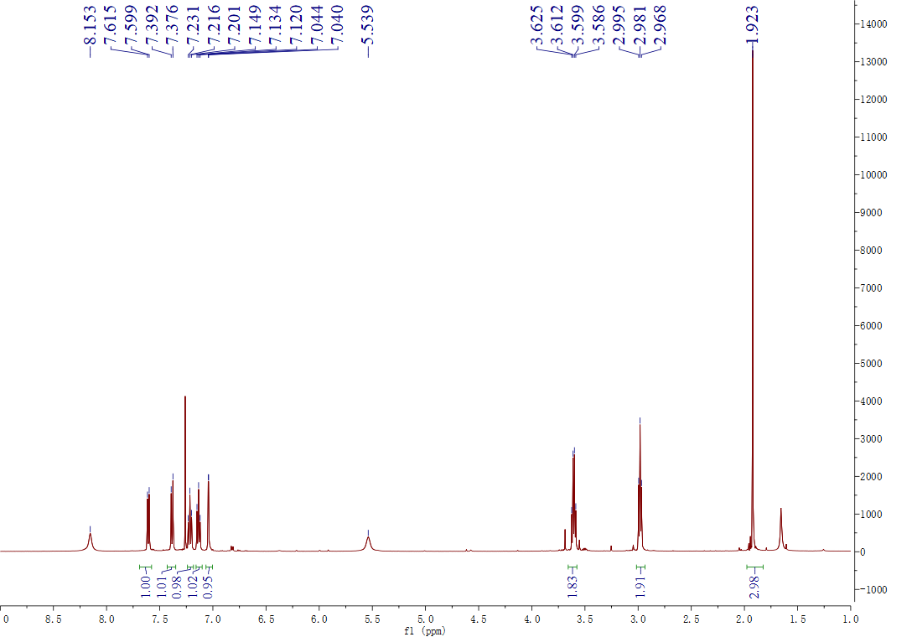


#### **Supplementary Figure 41** ^13^C NMR spectrum of **14** in CDCl_3_ (100MHz)


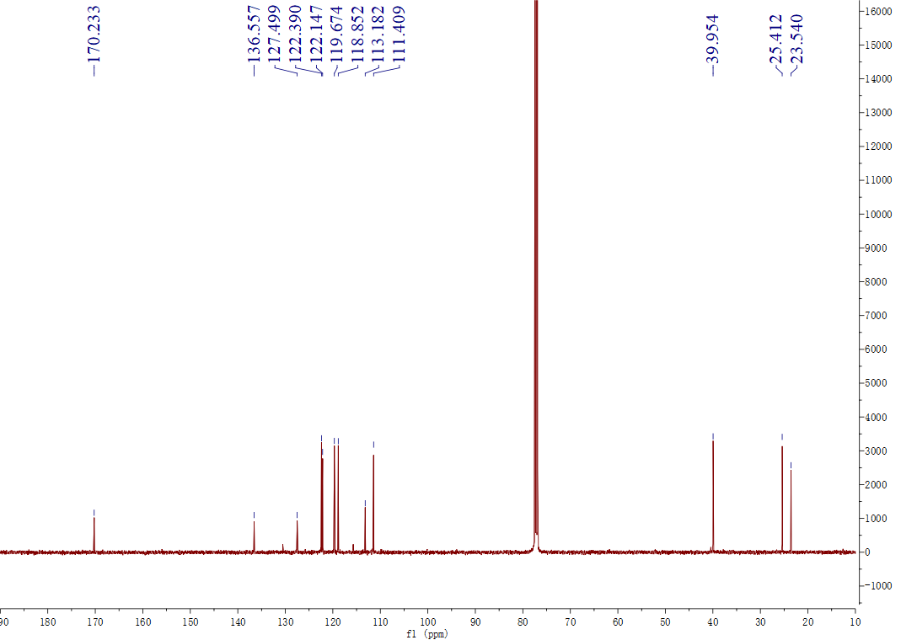


**Supplementary Figure 42** ^1^H NMR spectrum of **15** in CDCl_3_ (400MHz)


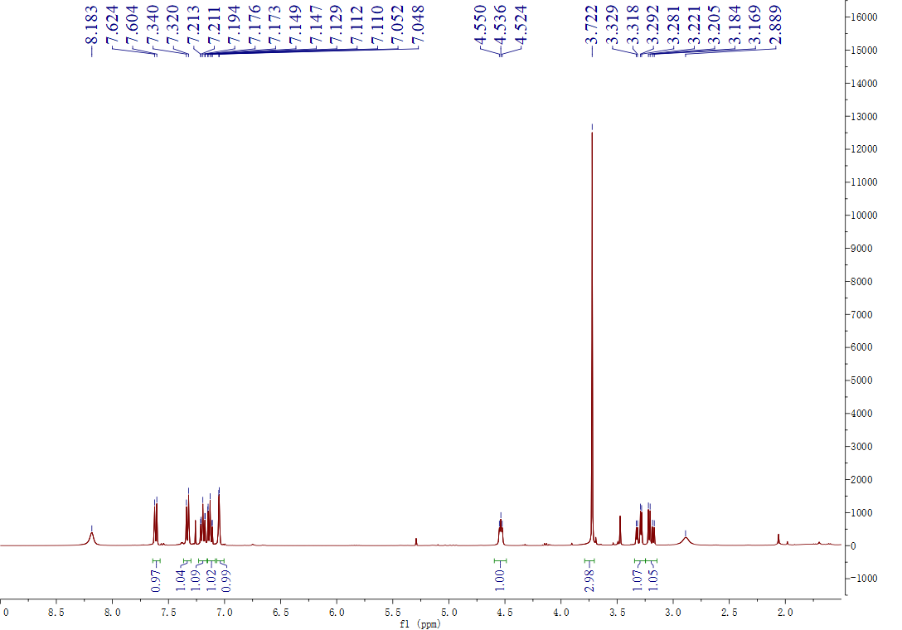


#### **Supplementary Figure 43** ^13^C NMR spectrum of **15** in CDCl_3_ (100MHz)


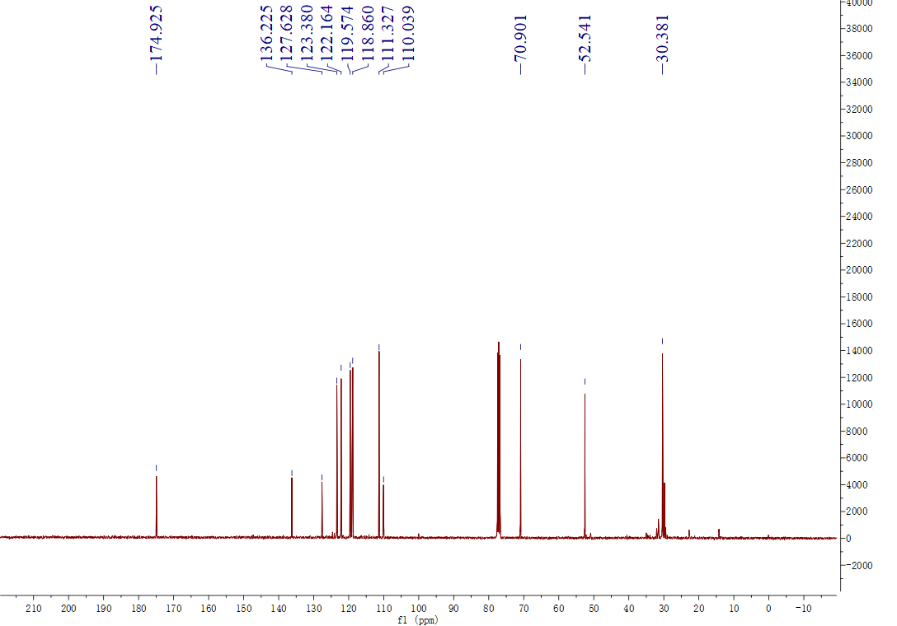


**Supplementary Figure 44** ^1^H NMR spectrum of **16** in acetone-*d*_6_ (400MHz)


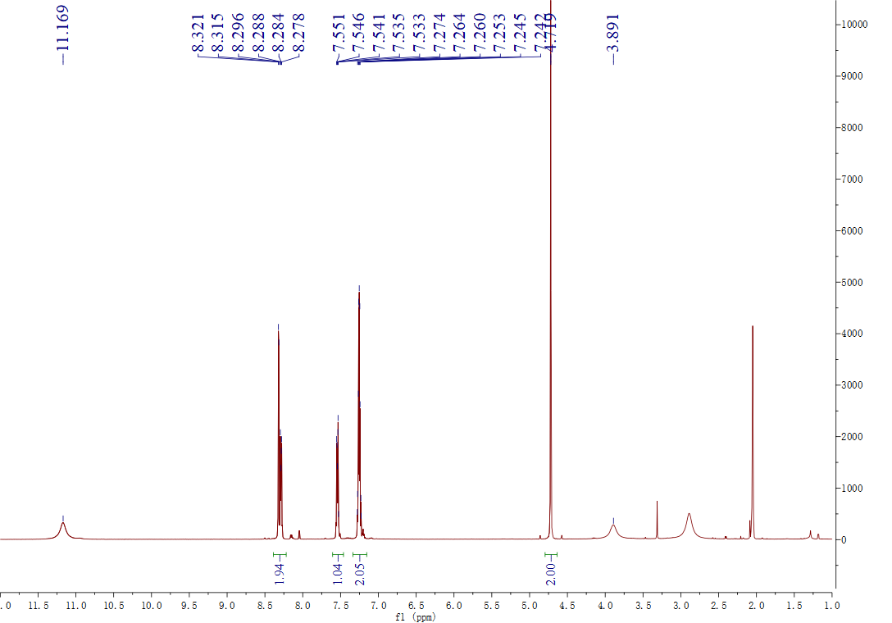


#### **Supplementary Figure 45** ^13^C NMR spectrum of **16** in acetone-*d*_6_ (100MHz)


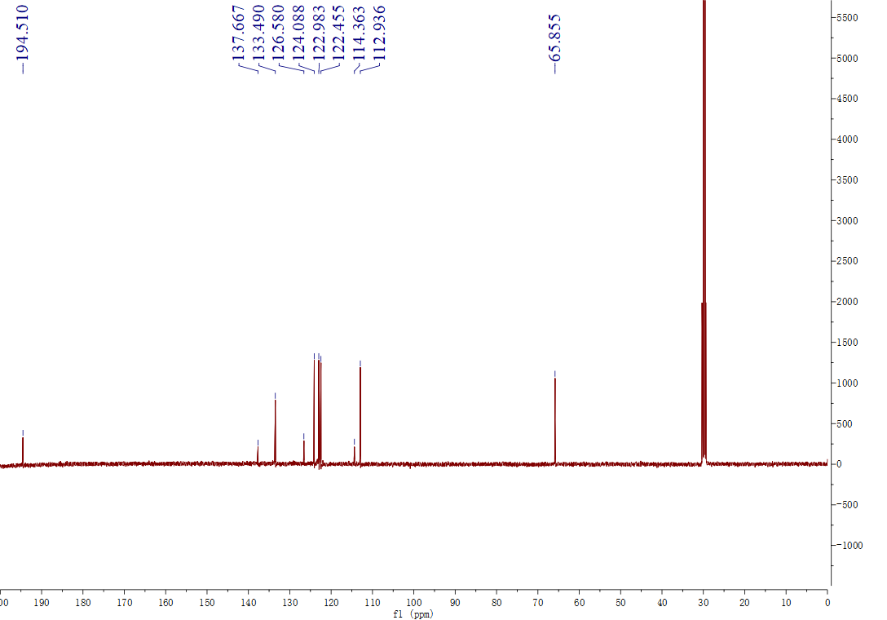


**Supplementary Figure 46** ^1^H NMR spectrum of **17** in acetone-*d*_6_ (400MHz)


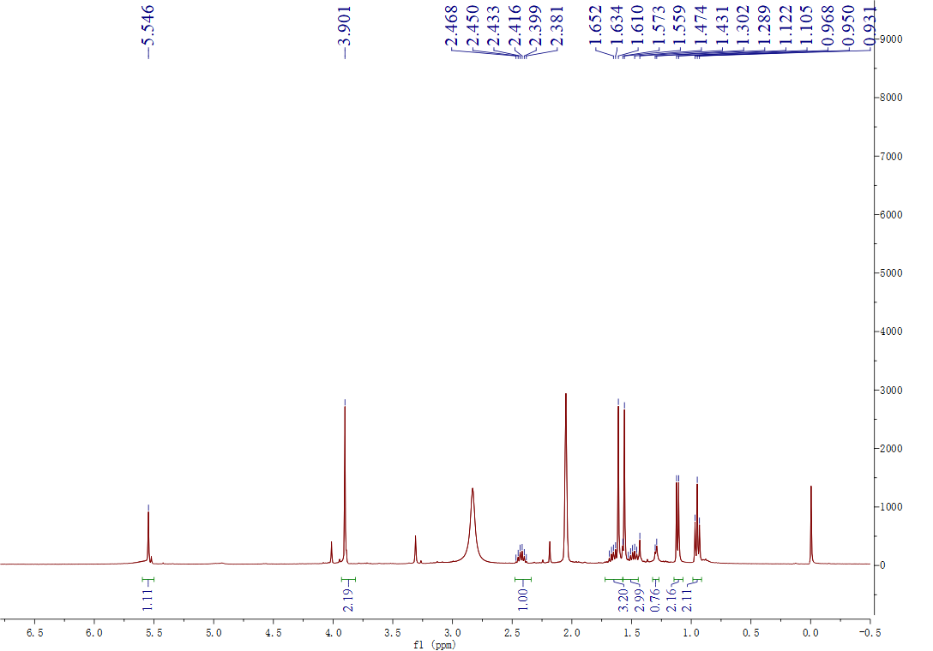


#### **Supplementary Figure 47** ^13^C NMR spectrum of **17** in acetone-*d*_6_ (100MHz)


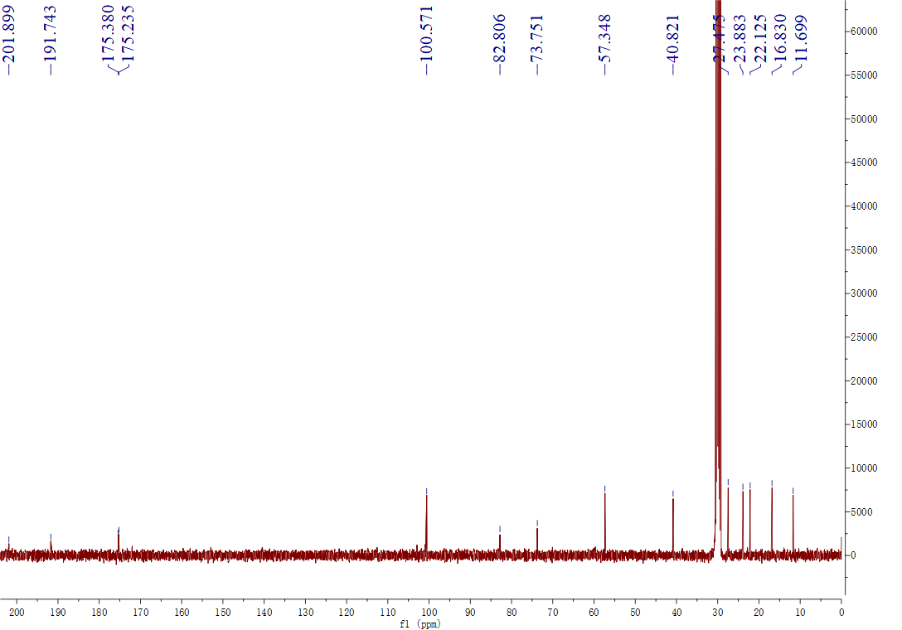


**Supplementary Figure 48** ^1^H NMR spectrum of **18** in methanol-*d*_4_ (400MHz)


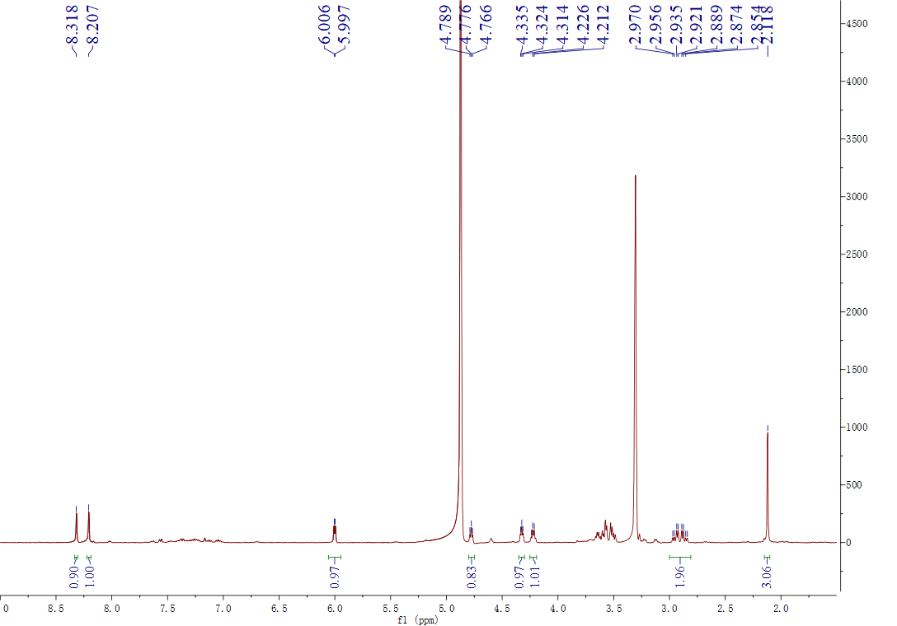


#### **Supplementary Figure 49** ^13^C NMR spectrum of **18** in methanol-*d*_4_ (100MHz)


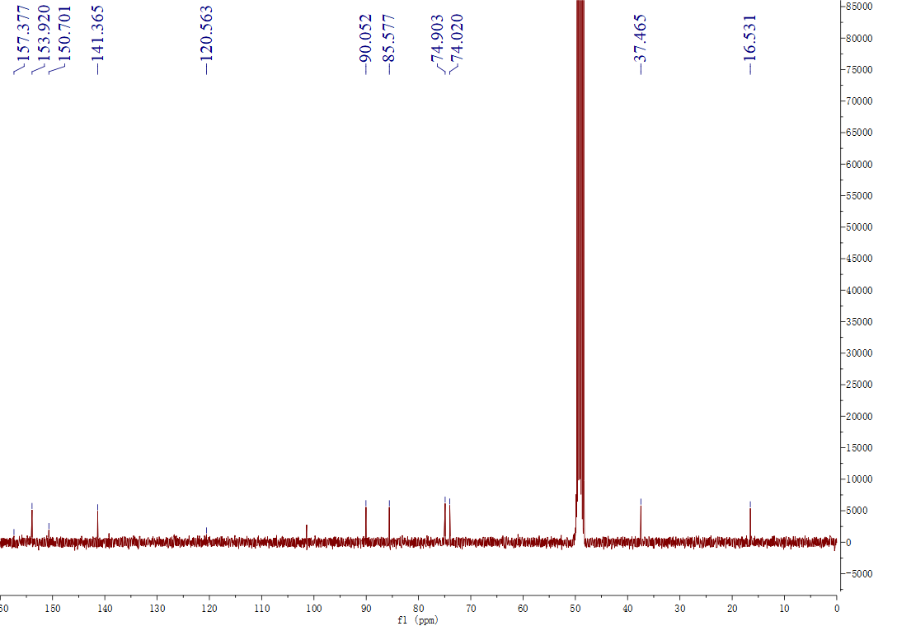


**Supplementary Figure 50** ^1^H NMR spectrum of **19** in acetone-*d*_6_ (400MHz)


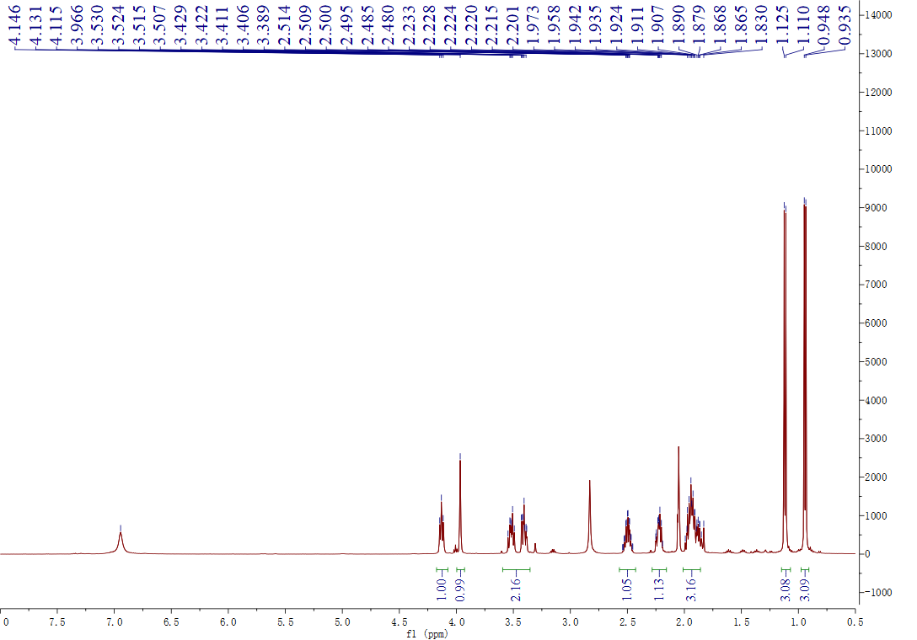


#### **Supplementary Figure 51** ^13^C NMR spectrum of **19** in acetone*-d*_6_ (100MHz)


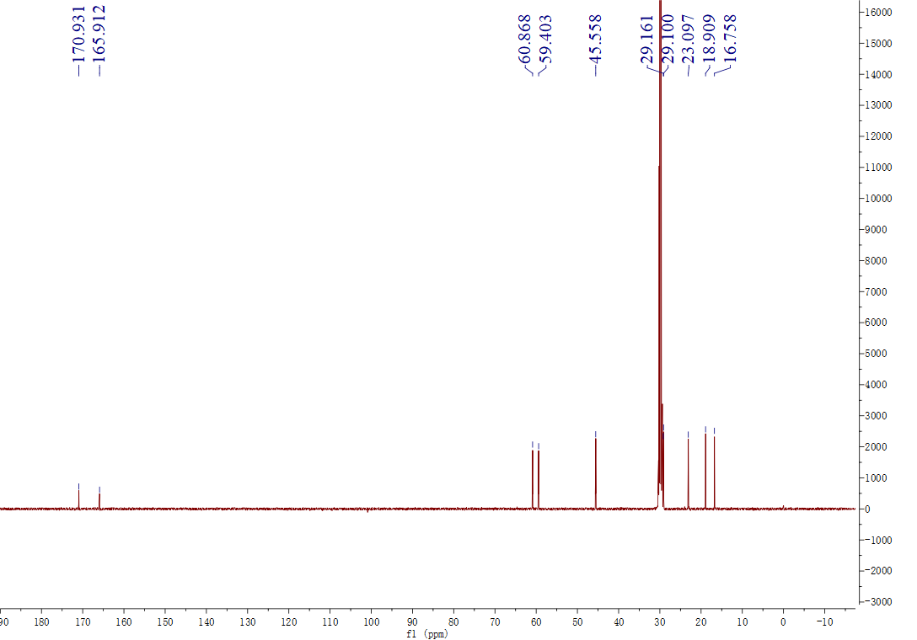


**Supplementary Figure 52** ^1^H NMR spectrum of **20** in acetone-*d*_6_ (400MHz)

####
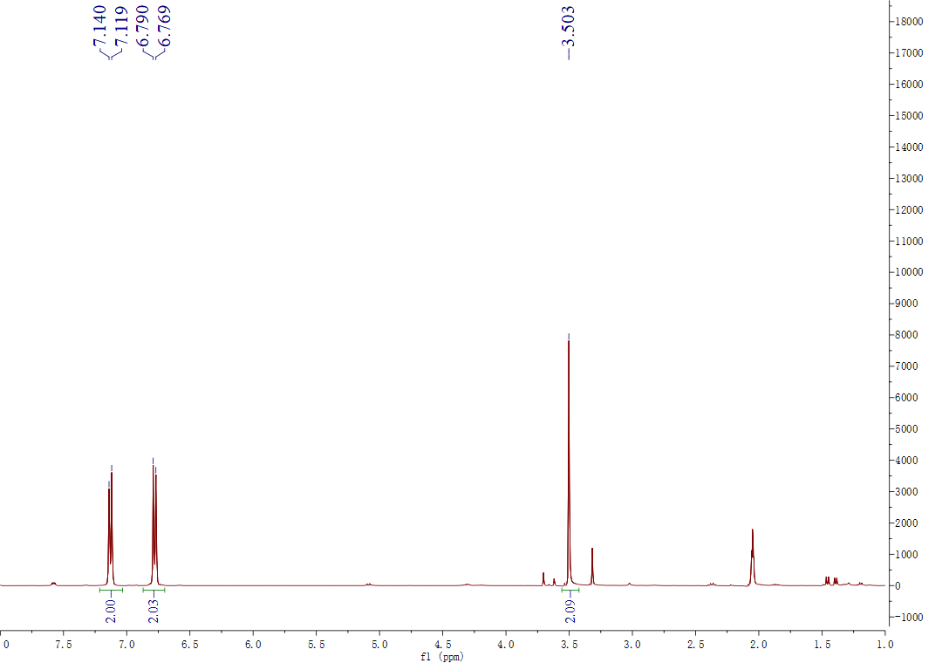
**Supplementary Figure 53** ^13^C NMR spectrum of **20** in acetone*-d*_6_ (100MHz)


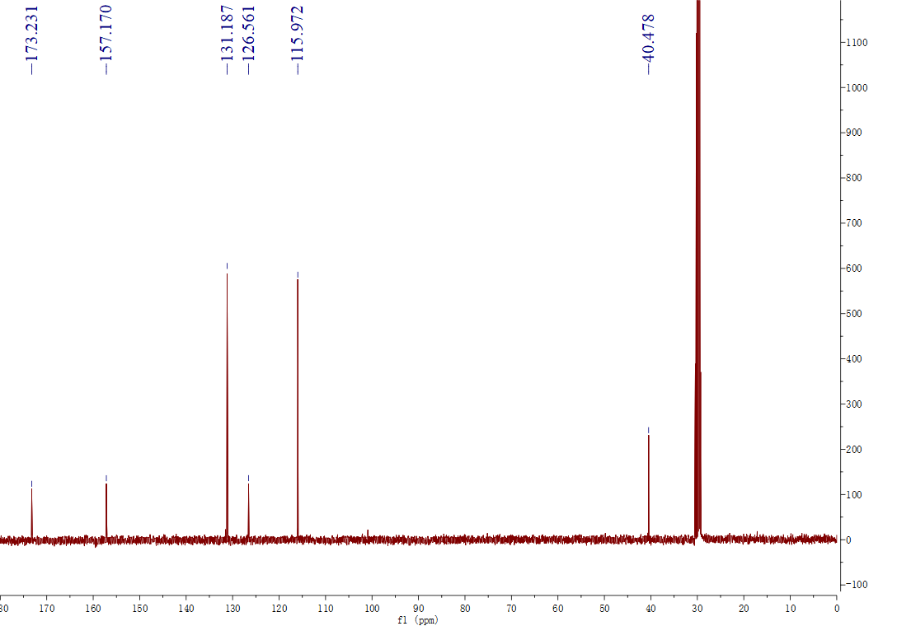


**Supplementary Figure 54** ^1^H NMR spectrum of **21** in acetone-*d*_6_ (400MHz)


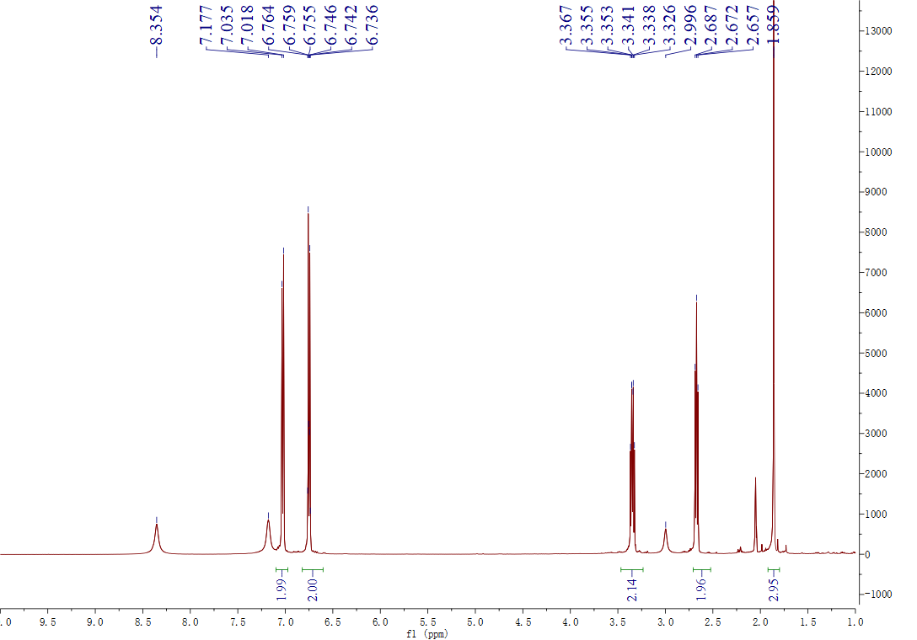


#### **Supplementary Figure 55** ^13^C NMR spectrum of **21** in acetone*-d*_6_ (100MHz)


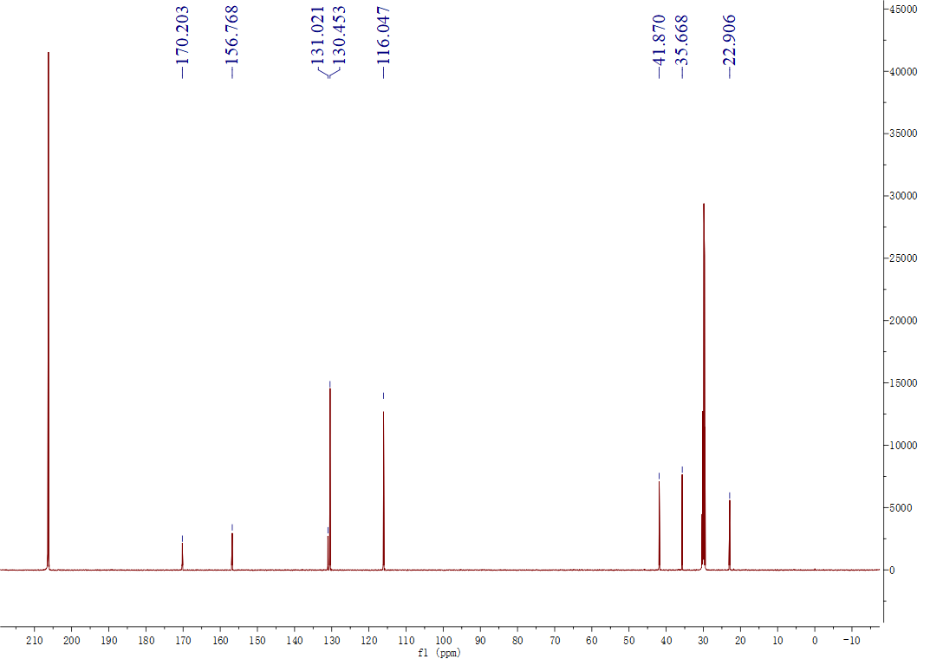


**Supplementary Figure 56** ^1^H NMR spectrum of **22** in CDCl_3_ (400MHz)


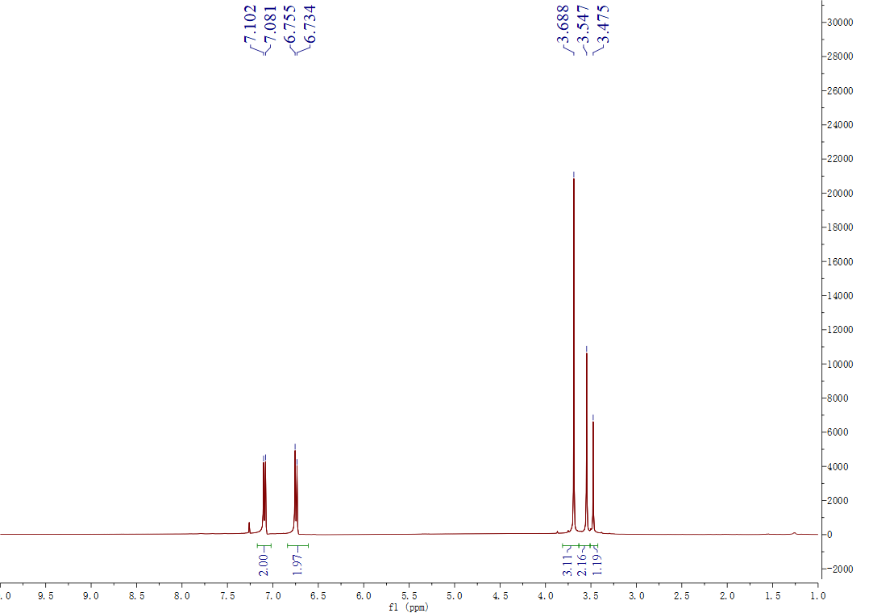


#### **Supplementary Figure 57** ^13^C NMR spectrum of **22** in CDCl_3_ (100MHz)


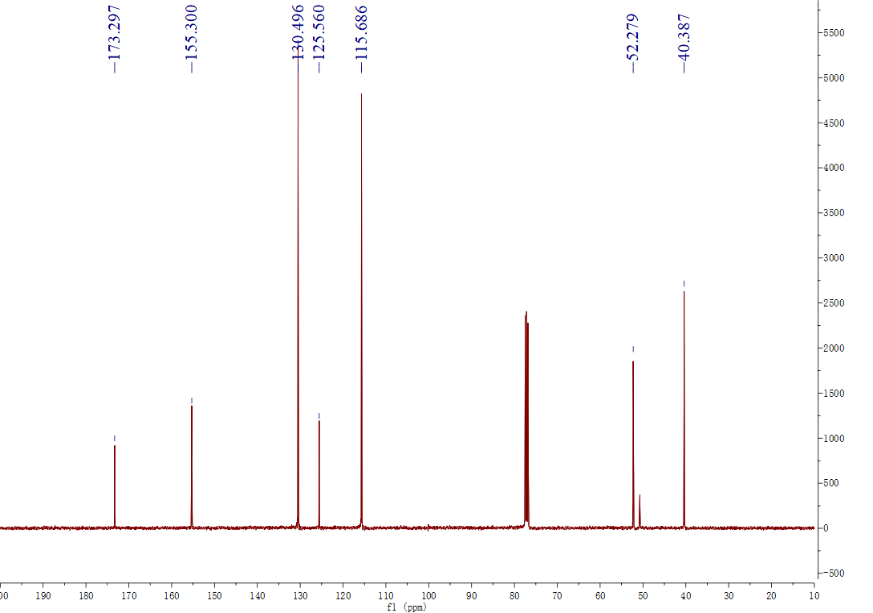


**Supplementary Figure 62** ^1^H NMR spectrum of **23** in methanol-*d*_4_ (400MHz)


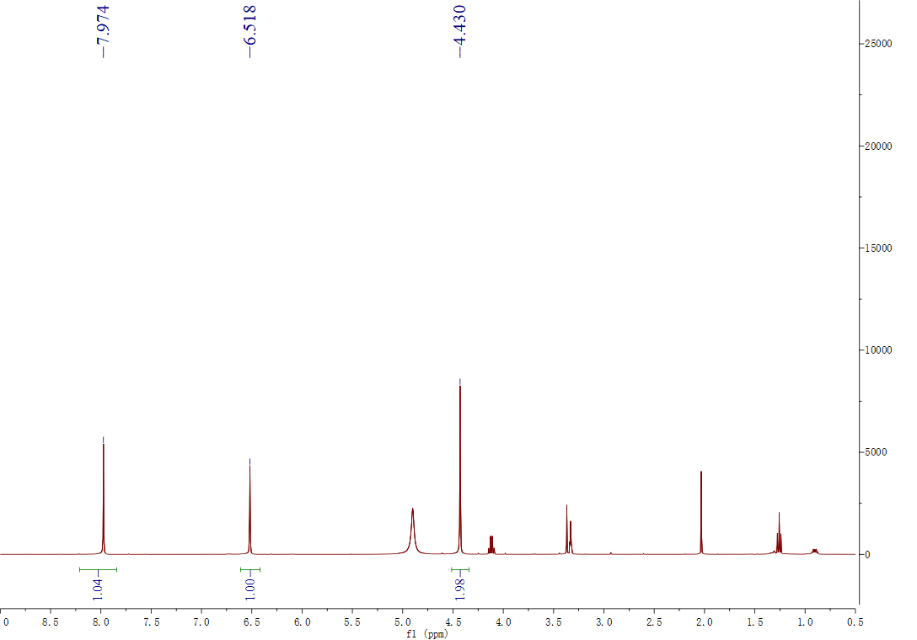


#### **Supplementary Figure 63** ^13^C NMR spectrum of **23** in methanol-*d*_4_ (100MHz)


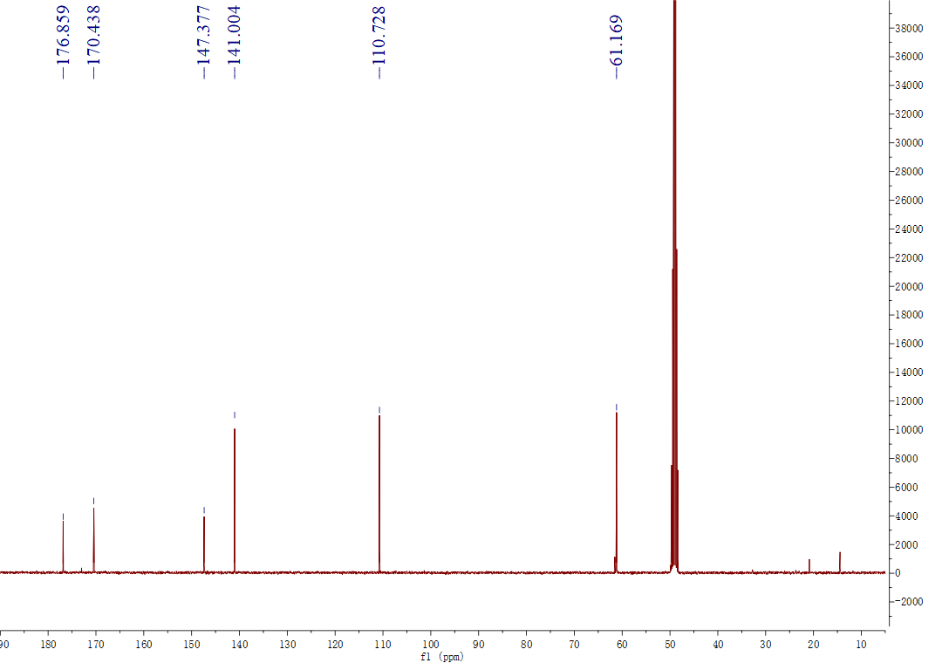


**Supplementary Figure 64** ^1^H NMR spectrum of **24** in DMSO-*d*_6_ (400MHz)


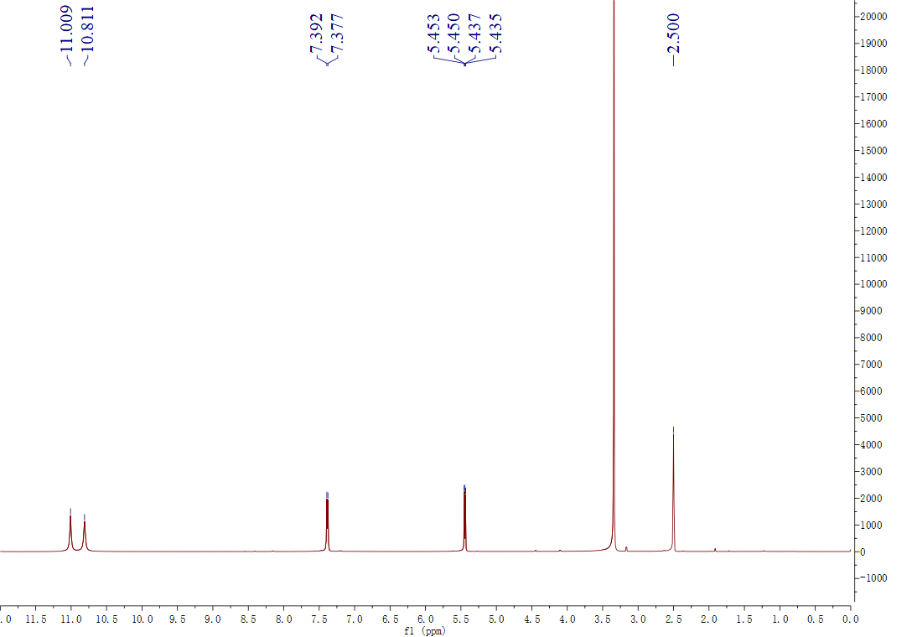


#### **Supplementary Figure 65** ^13^C NMR spectrum of **24** in DMSO*-d*_6_ (100MHz)


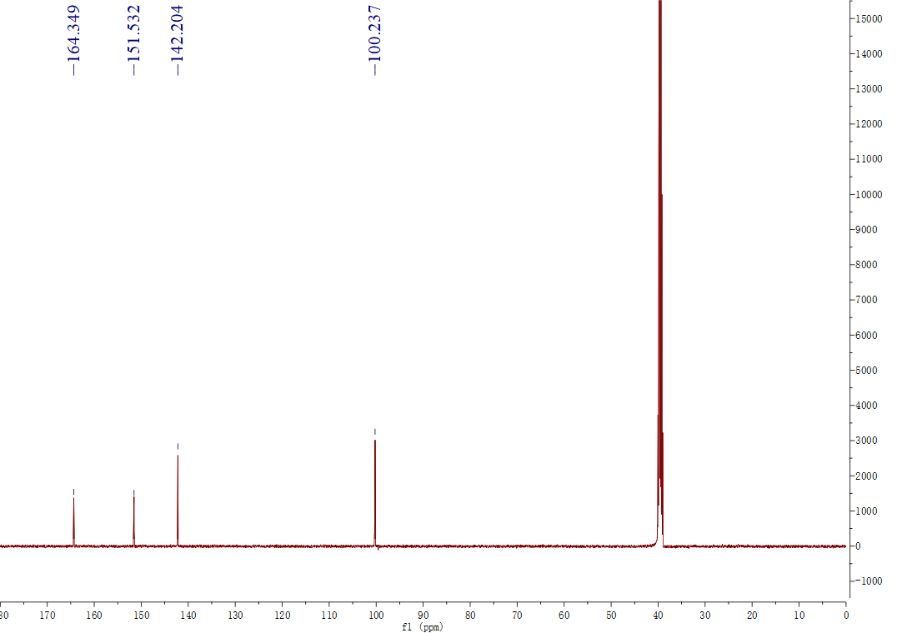


**Supplementary Figure 66** ^1^H NMR spectrum of **25** in methanol-*d*_4_ (400MHz)


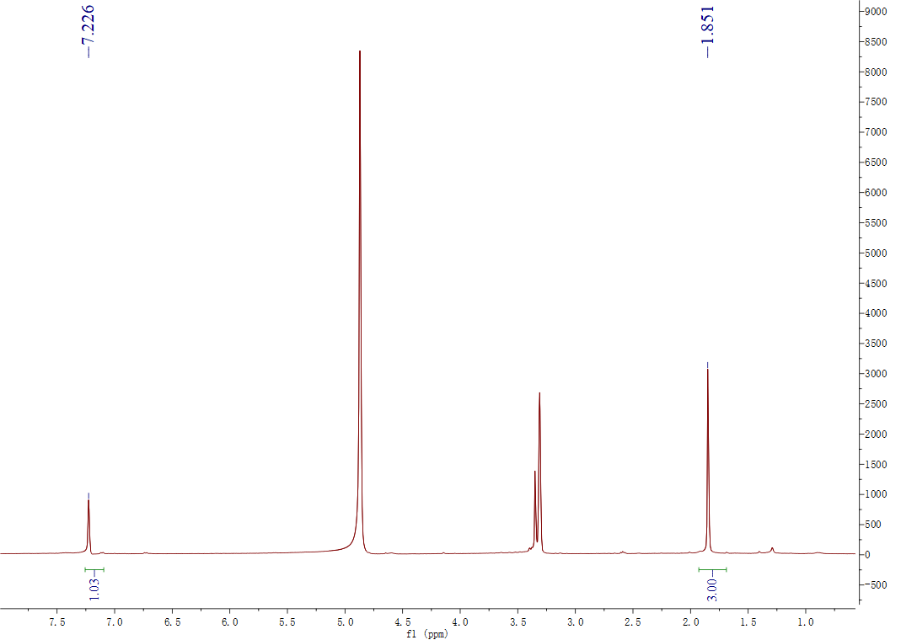


#### **Supplementary Figure 67** ^13^C NMR spectrum of **25** in methanol-*d*_4_ (100MHz)


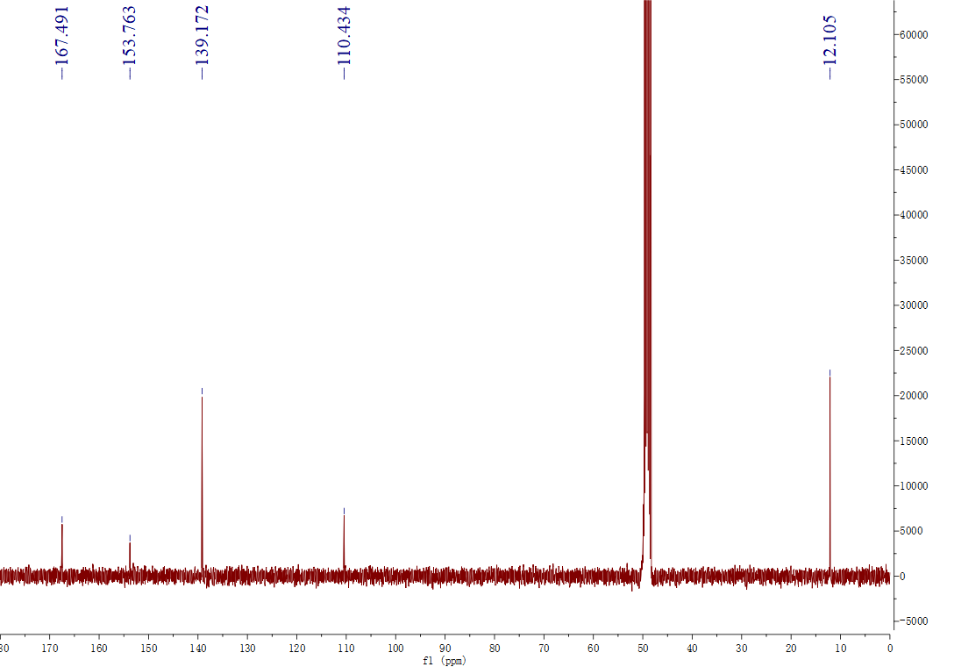

Supplement: Supplementary file 1 [file Data_Sheet_1.docx]
